# Near‐Armchair‐Enriched Growth of Single‐Walled Carbon Nanotubes via W‐Containing Fe‐Based Catalyst Systems in Mist FC‐CVD

**DOI:** 10.1002/smll.74316

**Published:** 2026-06-23

**Authors:** Hirotaka Inoue, Takamasa Onoki, Akira Takakura, Anastasios Karakassides, Hua Jiang, Toshihiko Fujimori, Esko I. Kauppinen

**Affiliations:** ^1^ Department of Applied Physics School of Science Aalto University Espoo Finland; ^2^ Sumitomo Electric Industries, Ltd. Osaka Japan; ^3^ Institute of Applied Physics University of Tsukuba Tsukuba Japan

**Keywords:** bimetallic catalyst, chiral angle distribution, floating catalyst chemical vapor deposition, mist CVD, single‐walled carbon nanotube

## Abstract

Chiral‐angle‐controlled synthesis of carbon nanotubes (CNTs) remains a central challenge, particularly in continuous growth processes such as floating catalyst chemical vapor deposition (FC‐CVD), where high productivity is attractive but tight structural control is difficult to achieve. In this study, a mist‐based FC‐CVD platform is established for continuous single‐walled CNT (SWCNT) growth, enabling systematic modulation of the chiral‐angle distribution through expanded catalyst‐precursor design. By introducing ultrasonic aerosol‐mist delivery, volatility constraints in precursor feeding are relaxed, allowing co‐delivery of a non‐volatile W precursor (ammonium metatungstate) with ferrocene to create a W‐containing Fe‐based catalyst system under atmospheric‐pressure FC‐CVD conditions. This catalyst system promotes pronounced near‐armchair enrichment, shifting the CNT chiral‐angle distribution away from achiral limits while narrowing the distribution compared with W‐free synthesis. Analysis based on a theoretical abundance expression for CNT growth on solid catalysts is consistent with a stabilization‐based interpretation, in which W‐containing species may increase the effective thermal/structural stability of Fe‐based catalyst nanoparticles and the catalyst–CNT interface, thereby strengthening chiral‐angle selectivity. Furthermore, tuning the carbon supply reveals an optimal regime for maximizing near‐armchair enrichment, whereas supply‐limited and overfed conditions broaden the distribution through distinct kinetic pathways. These results provide a practical route toward scalable chiral‐angle‐controlled CNT synthesis in continuous FC‐CVD.

## Graphical abstract

A mist‐based floating catalyst chemical vapor deposition (mist FC‐CVD) strategy allows the use of non‐volatile precursors and multi‐component catalyst delivery in a continuous process. Through catalyst composition control, it generates near‐armchair‐enriched single‐walled carbon nanotubes (SWCNTs) and uncovers key factors governing chiral‐angle selectivity, opening a practical pathway toward scalable and structure‐selective CNT synthesis.

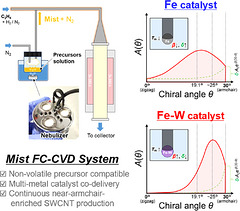

## 1 Introduction

Carbon nanotubes (CNTs) [1, 2] possess outstanding intrinsic properties, including high mechanical strength [3, 4], high thermal conductivity [5], superior electrical conductivity [6], and excellent chemical stability [7]. These properties can be preserved in CNT assemblies spanning dimensions and morphologies from nanoscale structures to macroscopic forms, enabling extensive development of CNT‐based materials for a broad range of applications, such as high‐strength fibers [8, 9], electrically conductive wires [10, 11], field‐effect transistors (FETs) [12, 13], flexible thermoelectric generators [11, 14, 15], ultrafast Joule heaters [16, 17], and thermal management materials [18, 19, 20, 21]. To enable practical deployment of these technologies, the ability to synthesize CNTs with well‐controlled structures is indispensable. Extensive efforts have therefore been devoted to structural control in CNT growth, including control over tube diameter [22, 23], number of walls [24, 25], tube length [26, 27, 28], and crystallinity [29, 30]. In addition, the selective synthesis of specific electronic types and structural distributions, such as semiconductor‐enriched single‐walled CNTs (SWCNTs) [31, 32, 33], metallic‐enriched SWCNTs [34, 35, 36, 37, 38], and CNT populations with narrow chiral‐angle distributions [39, 40], has attracted substantial interest. Elemental substitution doping, for example with nitrogen [41, 42] or boron [43, 44], has also shown notable progress.

Among these structural parameters, the chiral angle (*θ*) continues to attract particular interest because a range of structure‐dependent behaviors has been reported, especially for CNTs with chiral angles close to the armchair limit (*θ* ≈ 30°, near‐armchair). Armchair CNTs with *θ* = 30° are metallic, whereas ultra‐near‐armchair CNTs in which the armchair configuration is perturbed by one or two indices, for example, (n,n‐1) or (n,n‐2), are semiconducting [45]. In terms of mechanical properties, CNTs have also been experimentally shown to exhibit higher tensile strength as the chiral angle approaches the near‐armchair limit [46]. From the perspective of crystal growth, the chiral angle has been linked to CNT growth efficiency [35, 47] and defect density [48], and near‐armchair CNTs have been found to grow rapidly while maintaining relatively low defect densities. Furthermore, heat treatment below 1000°C can selectively coalesce same‐chirality (n,m) CNT pairs into (2n,2m) nanotubes while preserving the chiral angle, with particularly high efficiency for near‐armchair and armchair species (*θ* ≥ 25°), pointing to a bottom‐up route for structural control of larger‐diameter CNTs [49]. These experimentally and theoretically established near‐armchair‐specific behaviors provide sustained motivation for chiral‐angle control. Such control may enable further optimization of CNT performance and broaden the design space for future applications.

Chirality‐selective CNT synthesis has been widely explored in substrate‐based chemical vapor deposition (CVD), where catalysts can be precisely designed and tuned before growth. In particular, catalysts composed of two or more metals have enabled exceptionally high selectivity. For example, the W_6_Co_7_ bimetallic catalyst yielded CNTs with an overall metallic fraction of 95.5%, dominated by the (12,6) species (94.4% of all CNTs) [34], under one set of growth conditions. Under different growth conditions, the same catalyst yielded CNTs with an overall semiconducting fraction of 98.9%, dominated by the (14,4) species (97.4% of all CNTs) [50]. The remarkably high chirality selectivity achieved with W_6_Co_7_ has been attributed mainly to facet‐dependent structural templating, which provides a favorable match between the carbon arrangement around the nanotube circumference and the arrangement of catalytically active atoms on specific crystal planes, thereby promoting chirality‐defined nucleation and growth. In a similar catalyst design combining an iron‐triad metal (Fe, Co, and Ni) with a refractory metal, the CoRe_4_ bimetallic catalyst (bulk melting point ∼2000°C) has also been reported to yield metallic CNTs with ∼80% selectivity [37].

Beyond facet‐specific templating, the thermal and structural stability of catalyst nanoparticles has also emerged as an important design principle in CNT synthesis. In supported CVD systems, Fe‐ or Co‐based catalysts combined with refractory metals have been used to stabilize catalyst nanoparticles, suppress coarsening or Ostwald ripening, extend catalyst lifetime, and improve diameter or chirality control. For example, Fe/W mixed catalysts have been reported to extend Fe catalyst lifetime [51], Fe─Mo─W ternary catalysts can maintain catalyst size during temperature ramping and suppress agglomeration/Ostwald ripening [52], and Co─W alloy catalysts can retain solid crystalline structures under high‐temperature CVD conditions [34, 50]. These studies suggest that refractory‐metal‐assisted stabilization of Fe‐ or Co‐based catalysts can influence catalyst evolution and CNT structural selectivity. Together with the facet‐templating examples described above, these studies highlight that catalyst designs extending beyond conventional single‐metal catalysts based on the iron triad elements can substantially enhance the precision of structural control in CNT synthesis.

Floating catalyst chemical vapor deposition (FC‐CVD) is attractive for industrially relevant CNT production because it enables continuous production of highly crystalline CNTs in a high‐temperature gas‐phase environment [53]. Motivated by the high selectivity achieved in substrate‐based studies, several attempts have been made to introduce multi‐metal catalyst concepts into FC‐CVD. Chiang et al. generated Fe─Ni nanoparticles by decomposing ferrocene and nickelocene at a predefined mixing ratio using an atmospheric‐pressure microplasma, achieving continuous synthesis of CNTs enriched to more than 90% semiconducting species [54, 55]. Chauhan et al. demonstrated FC‐CVD using three metallocene precursors (ferrocene, nickelocene, and cobaltocene) to form Fe, Fe─Ni, Fe─Co, and Fe─Ni─Co catalyst systems [56], although the discussion in that work primarily focused on productivity rather than detailed structure selectivity. Moon et al. also employed mixed ferrocene and nickelocene precursors and reported that Ni addition promoted carbon feedstock decomposition, reduced the required growth temperature, and increased the growth rate. Although a quantitative chirality analysis was not performed, changes in the metallic and semiconducting fractions were inferred from radial breathing mode (RBM) analysis in Raman spectroscopy [57]. Overall, these studies indicate that multi‐metal catalyst strategies can offer important benefits in FC‐CVD. However, most demonstrations have been limited to metallocene‐derived catalysts and Fe/Co/Ni‐based combinations, while refractory metals such as W [34, 50] and Re [37] remain largely unexplored in FC‐CVD despite their demonstrated selectivity in substrate‐based CVD.

One major reason for this limited exploration is the restricted availability of catalyst precursors that satisfy the stringent requirements of conventional FC‐CVD delivery schemes. In typical FC‐CVD processes, catalyst precursors are delivered either by sublimating a solid precursor upstream and transporting the vapor into the growth zone for thermal decomposition, or by injecting a precursor solution, typically using a syringe pump, vaporizing it upstream, and carrying it into the growth zone. In both cases, the precursor must possess sufficiently high volatility (ideally from room temperature to ∼500°C) and suitable thermal decomposition characteristics (ideally well above room temperature yet below CNT growth temperatures, typically up to ∼1000°C). For solution‐based feeding, sufficient solubility in CNT‐compatible solvents such as toluene or ethanol is additionally required. When scale‐up is considered, precursor availability and cost also become important practical constraints. Because only a limited number of catalyst precursors satisfy these combined criteria, the precursor requirement itself narrows the accessible catalyst compositions and restricts the design space for multi‐metal FC‐CVD aimed at structure‐controlled CNT synthesis.

To relax these precursor constraints and expand catalyst design freedom in FC‐CVD, we integrated an ultrasonic aerosol‐mist delivery approach into the process, hereafter referred to as mist FC‐CVD. Ultrasonic nebulization can generate micrometer‐scale droplets from a precursor solution, allowing even low‐volatility or non‐volatile solutes to be transported into the reactor as suspended microdroplets by a carrier gas. By coupling this aerosol‐mist generation with thermal CVD, mist‐CVD has emerged as a simple and scalable route for forming high‐quality materials, including compositionally complex films and particulate deposits, without requiring vacuum‐based systems. Mist‐CVD has been extensively developed for oxide semiconductors and functional oxide materials, including wide‐bandgap oxides such as ZnO [58, 59] and Ga_2_O_3_ [60], superconducting films such as YBa_2_Cu_3_O_7‐_ *_x_* (YBCO) [61] and MgB_2_ [62], and halide perovskites such as CH_3_NH_3_PbX_3_ (X = Cl, Br, I) [63, 64]. In the CNT field, several studies have reported FC‐CVD growth assisted by ultrasonic aerosol‐mist supply [65, 66, 67]. However, these systems have mostly relied on single‐metal catalysts, and systematic investigations aimed specifically at overcoming precursor‐delivery limitations for multi‐metal FC‐CVD remain scarce.

In this study, we demonstrate chiral‐angle‐modulated CNT synthesis by introducing a W‐containing Fe‐based catalyst system into mist FC‐CVD. Ferrocene was used as the Fe precursor, while a non‐volatile W precursor, ammonium metatungstate, was dissolved in methanol with citric acid as a solubilizing and complexing agent, and the resulting solution was continuously delivered into the reactor as an ultrasonic aerosol mist. The produced CNTs were systematically characterized, including precise chirality analysis based on nanobeam electron diffraction (NBED). We found that introducing W‐containing species reduces CNT diameter and shifts and narrows the chiral‐angle distribution toward the near‐armchair region. We also reveal a strong dependence of the chiral‐angle distribution on the ethylene (C_2_H_4_) flow rate, and provide a mechanistic interpretation by linking the results to existing theories of CNT growth. This approach expands the accessible catalyst precursor space in FC‐CVD and provides a practical route for exploring refractory‐metal‐containing catalysts for advanced structure‐controlled CNT synthesis.

## 2 Results and Discussion

### 2.1 Structural Control of CNTs Grown With W‐Containing Fe‐Based Catalyst Systems in Mist FC‐CVD

**FIGURE 1:**
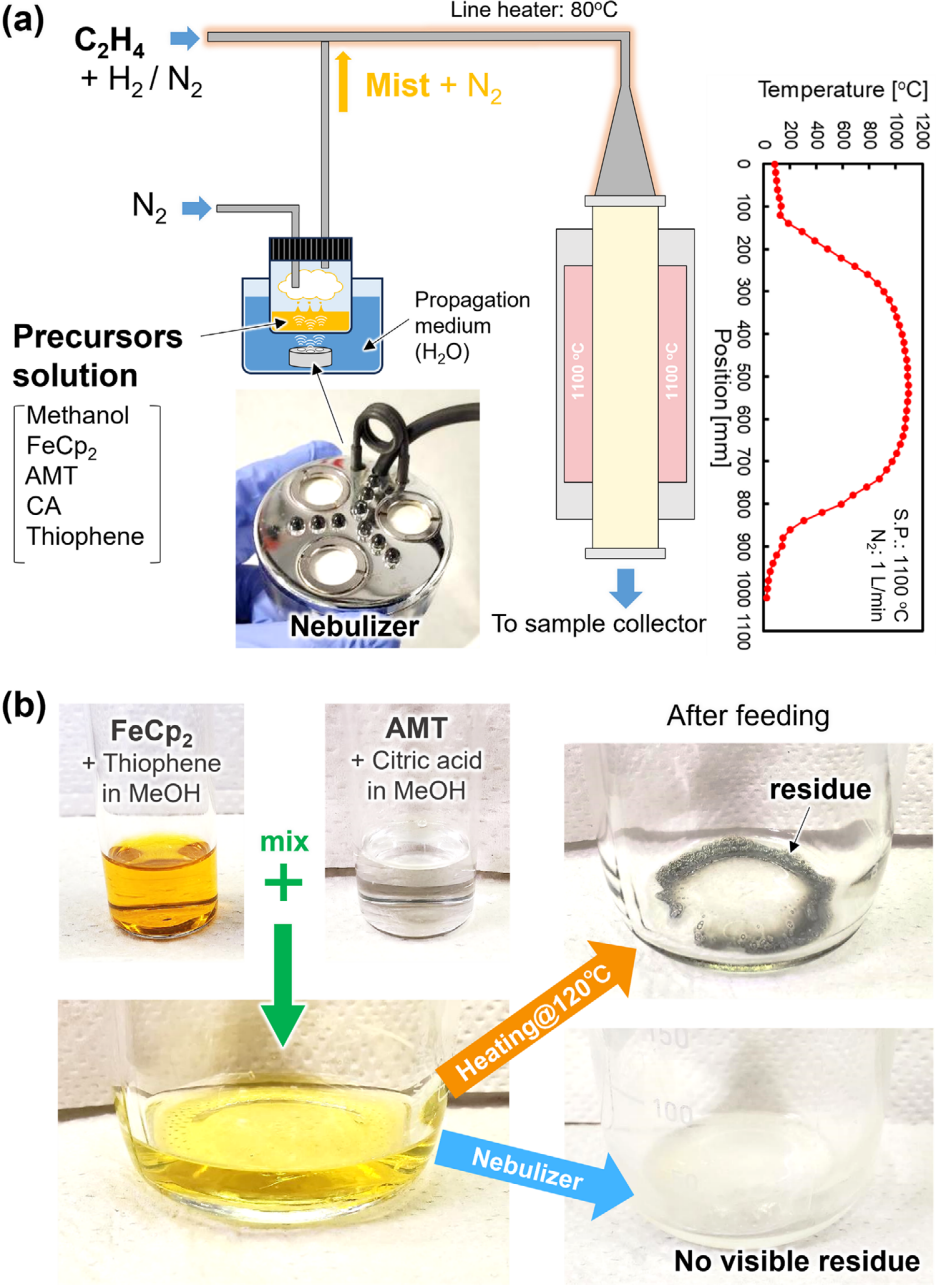
(a) Schematic of the FC‐CVD reactor equipped with an ultrasonic nebulization unit for mist‐based precursor delivery (mist FC‐CVD). (b) Photographs of the precursor solutions before and after mixing, and of the precursor container after all visible liquid had disappeared under two continuous precursor‐delivery conditions: hot‐plate heating at 120°C (heating‐based delivery) and ultrasonic nebulization (mist feeding).

We first verified whether both the Fe precursor (ferrocene) and the W precursor (ammonium metatungstate hydrate: AMT) were successfully delivered into the reactor using the designed mist FC‐CVD system (Figure 1a). Figure 1b compares photographs of the precursor container after all visible liquid had disappeared under two representative conditions: (i) continuous carrier‐gas flow with the container heated on a hot plate set to 120°C, representing a conventional liquid‐feed delivery scheme based on upstream heating and vaporization, and (ii) continuous carrier‐gas flow with ultrasonic nebulization to generate aerosol mist, corresponding to the mist‐feed condition used in this study. While a black residue remained in the container after hot‐plate heating, no visible residue remained when the precursor solution was supplied via aerosol mist generation. AMT has been reported to undergo thermal decomposition in an inert atmosphere through release of crystal water between 25°C and 200°C to form dehydrated AMT, formation of an amorphous phase between 200°C and 380°C, crystallization into hexagonal WO_3_ between 380°C and 500°C, and transformation into the more stable monoclinic WO_3_ between 500°C and 600°C [68]. Therefore, within the temperature range typically used for precursor vaporization in FC‐CVD, namely from room temperature to approximately 200°C, including the 120°C used here, AMT is unlikely to volatilize and be transported in vapor form. The residue observed after hot‐plate heating is thus most plausibly attributed to dehydrated AMT formed during this process. These results indicate that conventional heating‐based delivery is not suitable for supplying AMT into the reactor, whereas the mist‐feed approach enables such delivery.

**FIGURE 2:**
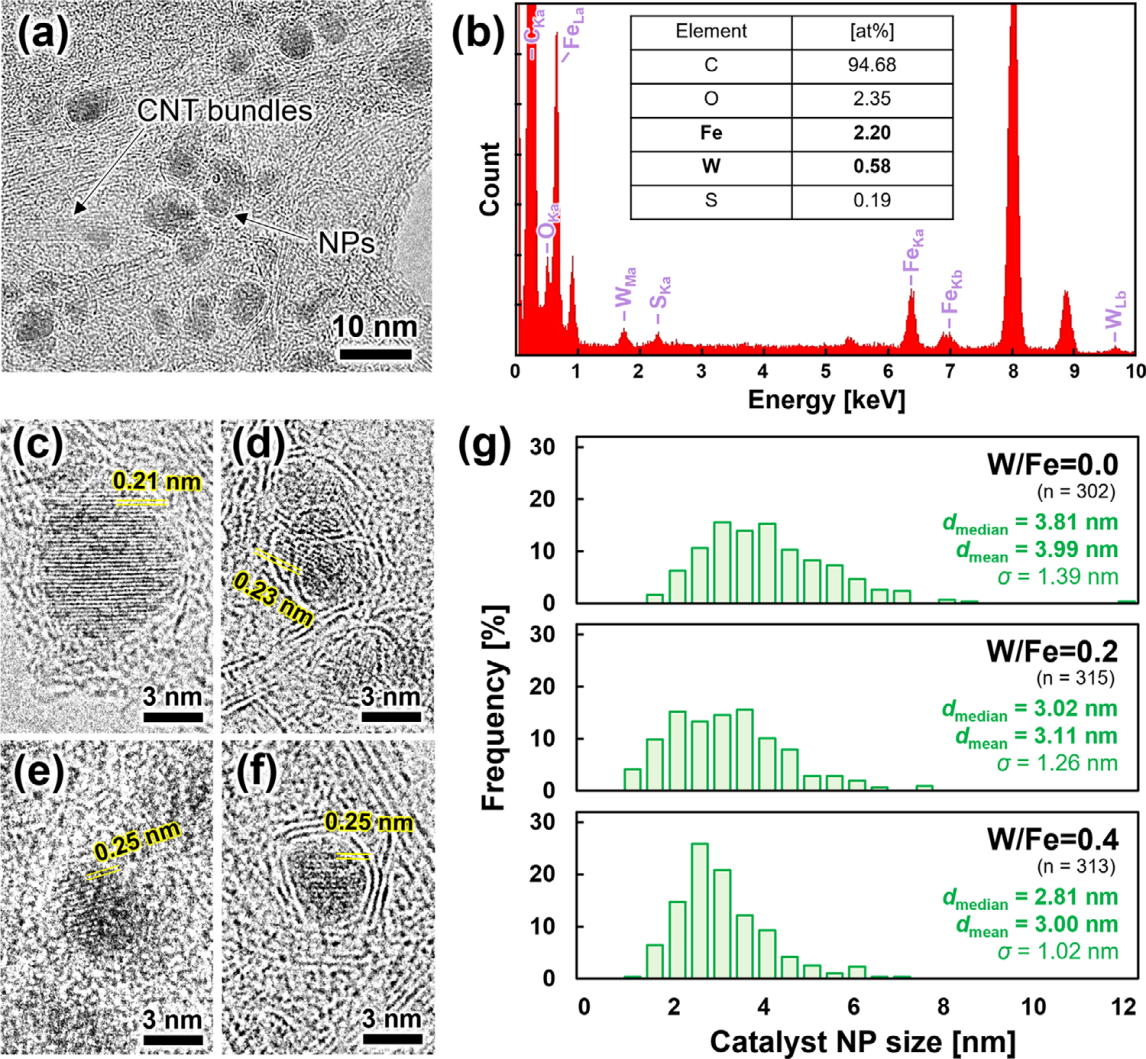
(a) Representative TEM image of CNTs and post‐growth catalyst‐related nanoparticles (NPs). (b) TEM‐EDS spectrum acquired from NPs. (c–f) HR‐TEM images of NPs highlighting lattice fringes. (g) Diameter distributions of NPs extracted from the HR‐TEM images.

To obtain direct evidence that W was transported into the reactor and reached the product stream, the materials collected on the filter after synthesis were examined by high‐resolution transmission electron microscopy (HR‐TEM). Figure 2a shows a representative TEM image of metal nanoparticles and CNT bundles, while additional representative post‐growth TEM images of CNT products synthesized at different W/Fe ratios are provided in Figure S1 as supplementary morphological information. HR‐TEM observations were also performed to examine the CNT wall number for the W/Fe = 0.0, 0.2, and 0.4 samples. Within the analyzed population of approximately 150 CNTs for each sample, no double‐walled or multi‐walled CNTs were observed, confirming that the examined CNTs were single‐walled. Figure 2b shows the energy‐dispersive X‐ray spectroscopy (EDS) spectrum acquired from the area shown in Figure 2a. In addition to the intense C and Fe signals, a clear W peak was detected, confirming the presence of W‐containing species in the collected products. For a representative sample prepared with a nominal precursor ratio of Fe:W = 1:0.4, the Fe:W atomic ratio estimated from EDS was 1:0.26.

In addition, HR‐TEM images of several metal nanoparticles exhibited lattice fringes with spacings of ∼0.21, 0.23, and 0.25 nm (Figure 2c–f), which are broadly consistent with Fe and/or Fe_3_C, as well as tungsten‐carbide‐related phases reported in the literature [25, 35, 69]. Although heating AMT or its citric‐acid complexes can lead to the formation of tungsten oxides [68, 70], no lattice fringes clearly corresponding to the larger spacings often reported for WO_3_, such as approximately 0.38 nm for the (020) [71] or (002) [72] planes, were identified within the present observation range. This is consistent with the possibility that W species generated at high temperature may have been reduced under the H_2_‐ and carbon‐containing environment and converted, at least partly, into W‐containing carbide‐related species. It is also noteworthy that, as shown in Figure 2g, the diameter distribution of the nanoparticles observed by HR‐TEM shifted toward smaller sizes upon W addition (W/Fe = 0.0: 4.0 ± 1.4 nm; W/Fe = 0.2: 3.1 ± 1.3 nm; W/Fe = 0.4: 3.0 ± 1.0 nm). This trend suggests that W addition influenced nanoparticle evolution in the reactor, potentially affecting processes such as particle growth and coarsening. However, phase identification based solely on individual lattice spacings remains limited and should not be regarded as definitive. The lattice‐fringe observations are therefore treated only as supporting evidence. Nevertheless, these observations are consistent with the distinct residue behavior observed for the two precursor‐delivery methods in Figure 1b and with the TEM‐EDS result in Figure 2b, both of which support the conclusion that the non‐volatile W precursor was introduced into the reactor by mist feeding. Together, these results further support the interpretation that the introduced W was present under conditions conducive to reduction and that at least a portion of it became available for possible interaction with Fe‐based catalyst nanoparticles during CNT growth.

**FIGURE 3:**
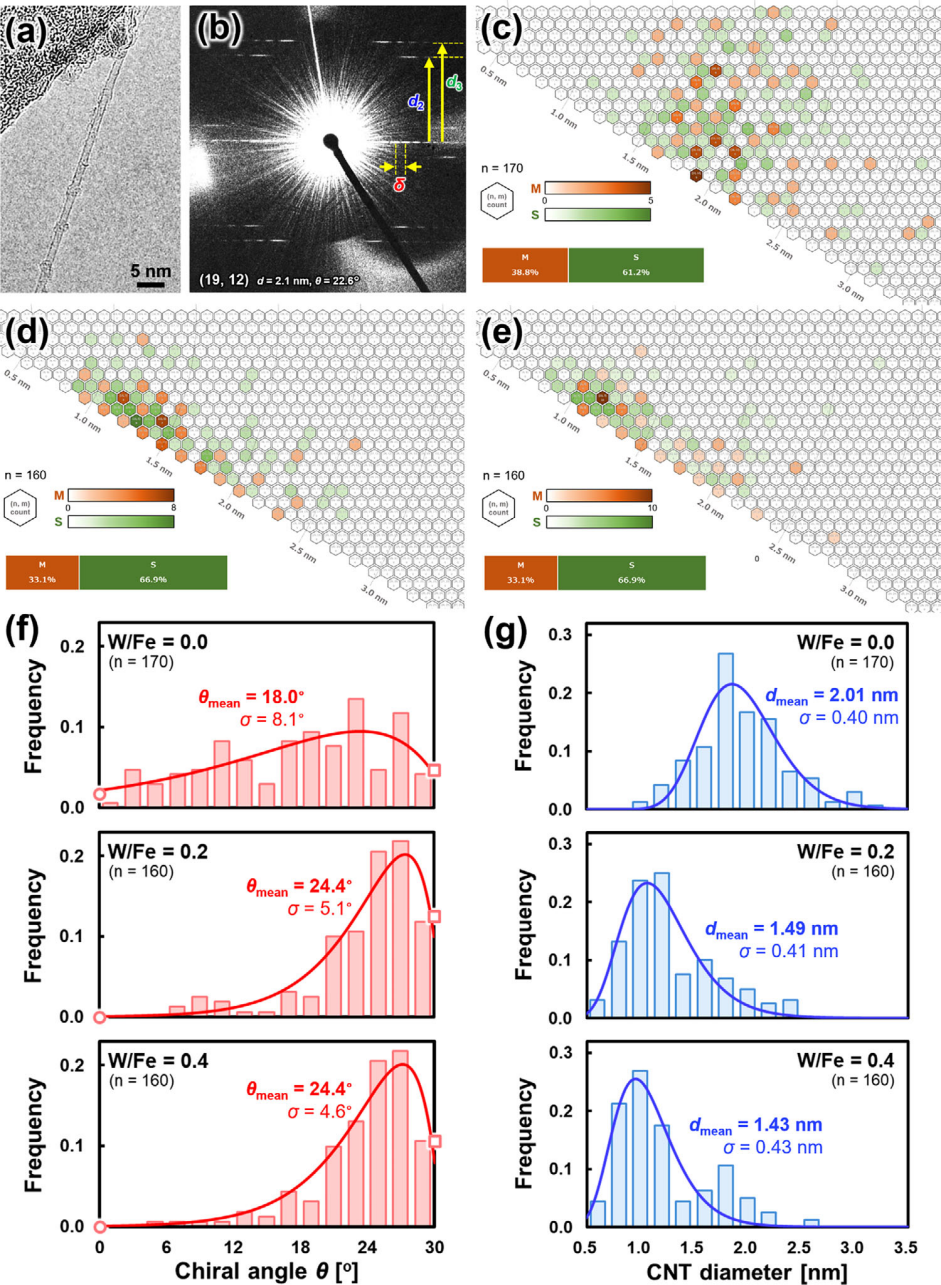
(a) Representative TEM image of an isolated SWCNT identified on the TEM grid for NBED analysis. (b) Corresponding NBED pattern acquired from the same SWCNT. (c–e) Chirality maps for W/Fe = 0.0, 0.2, and 0.4. (f) Chiral‐angle distributions extracted from the chirality maps (bin size: 2°). Red solid lines show fits using Equation 1. Magenta circle and square markers at *θ* = 0° and 30° represent the fractions of zigzag and armchair CNTs, respectively. (g) CNT diameter distributions extracted from the chirality maps for W/Fe = 0.0, 0.2, and 0.4. The distributions were fitted with log‐normal functions, shown as blue solid lines.

To quantitatively evaluate the chirality of the synthesized CNTs, we performed TEM‐NBED analysis on individual tubes. Isolated CNTs deposited on the TEM grid were first identified (Figure 3a), and a nanobeam was then focused onto each selected tube to acquire an electron diffraction pattern (Figure 3b). More than 160 diffraction patterns were collected for each sample, and the corresponding chirality indices (n,m) were assigned and plotted as chirality maps (Figure 3c–e). The metallic fraction was 38.8% for the W‐free sample and slightly decreased to ∼33% for the W‐containing samples. More pronounced changes were observed in the chirality distribution. Without W, CNTs were broadly distributed over chiral angles from 0 to 30° and diameters from 1 to 3.5 nm. In contrast, when W was introduced at W/Fe ratios of 0.2 and 0.4, the distribution shifted toward higher chiral angles closer to the armchair limit, indicating near‐armchair enrichment.

This trend is clearly reflected in the chiral‐angle distributions shown in Figure 3f. Here, the chiral‐angle distribution was constructed by summing the number of CNTs in 2°‐wide bins. However, the zigzag (θ = 0°, shown as a magenta circle marker) and armchair (θ = 30°, shown as a magenta square marker) species, which are achiral and exhibit distinct theoretical and experimental characteristics [47, 73, 74], were counted and plotted separately from the binned distributions. For W/Fe = 0.0, the distribution was relatively broad and centered at lower angles, with *θ*_median_ = 19.1°, *θ*_mean_ = 18.0°, and standard deviation *σ* = 8.1°. With the addition of W, the distributions became narrower and shifted to higher angles. For W/Fe = 0.2, *θ*_median_ = 25.7°, *θ*_mean_ = 24.4°, and *σ* = 5.1°, while for W/Fe = 0.4, *θ*_median_ = 25.1°, *θ*_mean_ = 24.4°, and *σ* = 4.6°.

The change in distribution shape was further supported by the skewness and excess kurtosis, which quantify distribution asymmetry and peak sharpness, respectively. For reference, both values are zero for a normal distribution. Negative skewness indicates an asymmetric distribution with a tail toward lower angles and a peak shifted toward the higher‐angle side, whereas positive excess kurtosis indicates a sharper, more narrowly concentrated peak than that of a normal distribution. The W‐free sample exhibited skewness = −0.45 and excess kurtosis = −0.79, consistent with a relatively broad distribution. In contrast, W/Fe = 0.2 (skewness = −1.59, excess kurtosis = 2.57) and W/Fe = 0.4 (skewness = −1.40, excess kurtosis = 2.59) showed markedly sharper distributions biased toward high chiral angles (Table S1).

To further evaluate whether these apparent changes were statistically supported and not merely a consequence of histogram binning, we performed pairwise nonparametric tests using the raw chiral‐angle values (Tables S2 and S3). Mann–Whitney tests showed that the W‐containing samples were significantly shifted toward higher chiral angles relative to the W‐free sample (W/Fe = 0.2 vs. 0.0: *p* < 0.0001; W/Fe = 0.4 vs. 0.0: *p* < 0.0001), whereas no significant rank‐based shift was detected between W/Fe = 0.2 and 0.4 (*p* = 0.5924). The direction of the shift was determined from the mean ranks. Two‐sample Kolmogorov–Smirnov (KS) tests also confirmed that the overall empirical chiral‐angle distributions of the W‐containing samples differed significantly from those of the W‐free sample (W/Fe = 0.2 vs. 0.0: *p* < 0.0001; W/Fe = 0.4 vs. 0.0: *p* < 0.0001), while no significant distributional difference was detected between W/Fe = 0.2 and 0.4 (*p* = 0.5999). These conclusions remained unchanged after Bonferroni correction for three pairwise comparisons.

Figure 3g summarizes the effect of W addition on CNT diameter distributions. Consistent with several previous reports [75, 76, 77], the diameter distributions were well fitted by log‐normal functions. The median diameters *d*_median_ for W/Fe = 0.0, 0.2, and 0.4 were 1.96, 1.21, and 1.07 nm, respectively. The corresponding mean diameters *d*_mean_ were 2.01, 1.49, and 1.43 nm, and the standard deviations *σ* were 0.40, 0.41, and 0.43 nm. This trend toward smaller CNT diameters obtained from the TEM‐NBED analysis was further supported by Raman and UV–Vis–NIR optical characterization (Figures S2 and S3). Both the Raman RBM features and UV–Vis–NIR absorption spectra showed changes consistent with the NBED‐derived diameter reduction. These results show that the introduction of W‐containing species is associated with a statistically supported shift of the chiral‐angle distribution toward higher chiral angles near the armchair limit while simultaneously reducing CNT diameters.

To gain insight into the mechanism underlying the observed shift in chiral‐angle distributions, we analyzed the data using the theoretical framework proposed by Artyukhov et al. [47] for SWCNT growth on catalysts. In this framework, the abundance of CNTs with chiral angle *θ*, *A*(*θ*), is described as the product of the nucleation probability *N*(*θ*) and the growth rate *R*(*θ*), such that *A*(*θ*) ∝ *N*(*θ*)·*R*(*θ*). The nucleation term *N*(*θ*) is treated as a thermodynamic factor governed by the chiral‐angle dependence of the catalyst–CNT interfacial free energy, and therefore becomes larger as the chiral angle approaches an achiral endpoint. In contrast, the growth‐rate term *R*(*θ*) is a kinetic factor controlled by the density of kinks (active sites) at the CNT growth front. Within the solid‐catalyst framework of this model, *R*(*θ*) is predicted to peak near *θ* ≈ 19.1°, corresponding to the so‐called “magic angle” associated with (2m,m) chiralities [78, 79]. In the original formulation, the angular dependence is expressed in terms of the deviation *x* from the nearest achiral direction, where *x* = *θ* on the zigzag side and *x* = 30°−*θ* on the armchair side. Because the present samples exhibited a clear armchair‐side preference, we adopted *x* = 30°−*θ* and used the corresponding simplified form of the chiral‐angle‐dependent abundance:

(1) $$A\left(\theta \right)=A_{0}\;\left[\left(30^{\circ }-\theta \right)+\delta \right]\cdot e^{-\beta \left(30^{\circ }-\theta \right)}.$$

**FIGURE 4:**
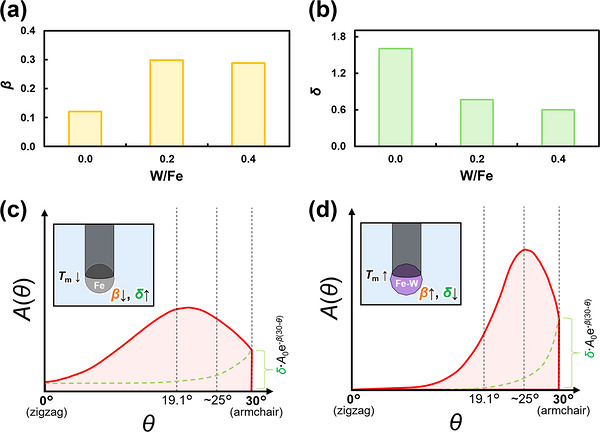
Fitting parameters (a) *β* and (b) *δ* extracted from Equation 1 for different W/Fe ratios. Schematic illustrations of changes in the chiral‐angle distribution for (c) Fe monometallic catalysts and (d) W‐containing Fe‐based catalyst systems. *T*_m_ schematically represents the effective thermal stability of the catalyst system.

Here, *A*_0_ is a scale factor, *β* represents the strength of the exponential decay with respect to (30°−*θ*) and thus quantifies the degree of angle selectivity, and *δ* is an offset term that accounts for baseline contributions near the achiral limit (see Supporting Information for the detailed derivation of the fitting expression). The fitting results obtained using Equation 1 are summarized in Table S1 and Figure 4a,b. Notably, *β* increased with W addition from 0.12 (W/Fe = 0.0) to 0.30 (W/Fe = 0.2) and 0.29 (W/Fe = 0.4), while *δ* decreased from 1.60 (W/Fe = 0.0) to 0.76 (W/Fe = 0.2) and 0.60 (W/Fe = 0.4).

The W‐induced changes in CNT structure are likely associated with modifications of the catalyst state in the reactor arising from the introduction of W‐containing species and their possible interaction with Fe‐based catalyst nanoparticles. Such interaction may alter both the physicochemical state of the catalyst particles present in the FC‐CVD reaction zone and the properties of the catalyst–CNT interface. Within the framework proposed by Artyukhov et al. [47], the nucleation probability *N*(*θ*) is exponentially weighted by the chiral‐angle dependence of the catalyst–CNT interfacial free energy. The strength of this angular weighting is described by the parameter *β*, which scales as *β* ∝ π*dγ*′/*k*_B_ *T*, where *d* is the CNT diameter and *γ*′ is an interfacial‐state parameter reflecting the chiral‐angle dependence of the catalyst–CNT interfacial free energy. In our experiments, the set growth temperature and carrier‐gas flow rates, which influence the thermal history of catalyst particles traveling through the reactor, were kept constant across samples. Therefore, *T* can be treated as a constant in the present analysis, and the observed variation in *β* can be attributed primarily to changes in *d* and *γ*′. If *d* were the dominant factor determining *β*, then the W‐induced decrease in *d* observed in this study (Figure 3g) would be expected to decrease *β* according to the relation *β* ∝ π*dγ*′/*k*_B_ *T*. However, the fitting results clearly showed the opposite trend, namely an increase in *β*, upon W addition. This indicates that the observed increase in *β* is governed primarily by an increase in *γ*′. For a simplified comparison, we therefore evaluated *β* normalized by the mean diameter *d*_mean_, namely *β*/*d*_mean_, which is proportional to *γ*′ under constant‐temperature conditions. The resulting values increased markedly from 0.06 (W/Fe = 0.0) to 0.20 (W/Fe = 0.2) and 0.20 (W/Fe = 0.4), suggesting a clear change in the catalyst–CNT interfacial state upon W addition. In this model, *γ*′ represents the energetic penalty associated with disrupting the “tight contact” at the catalyst–CNT interface. When the interface is more liquid‐like, it can rearrange readily during kink incorporation, and the chiral‐angle dependence of this penalty is less strongly reflected in the final distribution. In contrast, when the interface is more solid‐like and structure‐preserving, such rearrangement is restricted, and the chiral‐angle dependence becomes more pronounced. Within this stabilization‐based interpretation, introducing W‐containing species into the Fe‐based catalyst system may make the effective catalyst–CNT interfacial state more solid‐like, strengthen interfacial‐origin selectivity (higher *β*), and shift the distribution toward the near‐armchair side, consistent with Figure 4c,d.

The parameter *δ* also exhibited a clear dependence on W addition, decreasing from 1.60 for W/Fe = 0.0 to 0.76 for W/Fe = 0.2 and 0.60 for W/Fe = 0.4 (Figure 4b). According to Ref. [47], the chiral‐angle dependence of the growth rate includes not only the geometric kink contribution associated with screw dislocations [73], but also an additional contribution from fluctuational kinks that can form near the achiral boundaries (armchair and zigzag) through thermal fluctuations. These fluctuational kinks contribute additively to the growth rate and allow non‐negligible growth near achiral configurations that cannot be fully captured by the idealized geometric term alone. In our system, the decrease in *δ* upon W addition can be interpreted within this framework. If W‐containing species increase the effective thermal/structural stability of the catalyst particles and/or the catalyst–CNT interface, the formation of thermally activated fluctuational kinks is expected to be suppressed. As a result, the additive contribution that enhances growth near the achiral limits becomes less significant, leading to a reduced *δ* and a diminished baseline enhancement in the achiral vicinity.

The observed reduction in CNT diameter may also be interpreted within a catalyst‐stabilization framework. There is a temperature gap between precursor decomposition and the onset of CNT growth: ferrocene decomposes at approximately 400°C–500°C [80, 81, 82] and AMT decomposes at around 500°C [68], whereas CNT growth typically initiates above ∼800°C. During transport through the temperature‐gradient region of the reactor (Figure 1a), catalyst nanoparticles are likely to undergo frequent collisions, which can promote aggregation and coarsening before they reach the effective growth zone. If W‐containing species interact with Fe‐based nanoparticles and increase the effective thermal/structural stability of the catalyst particles, such coalescence may be suppressed, resulting in smaller particle sizes at the onset of CNT growth. This interpretation is consistent with the nanoparticle size distributions observed in Figure 2g. Although the post‐growth particle‐size distribution does not directly represent the active particle size at nucleation [83, 84], the observed shift toward smaller post‐growth catalyst‐related nanoparticles (Figure 2g) is consistent with reduced coarsening in the reactor. Because catalyst particle size is known to correlate strongly with CNT diameter across various synthesis approaches [75, 85], particularly at the onset of CNT growth [83, 86], the reduced CNT diameters observed here (Figure 3g) may reasonably be attributed, at least in part, to the suppression of catalyst coarsening, leading to smaller W‐containing Fe‐based catalyst‐related nanoparticles at the onset of CNT growth.

In addition, the shift in the nanoparticle size distribution toward smaller diameters (Figure 2g) may increase the fraction of particles that fall within the size range favorable for SWCNT growth, while reducing the fraction of oversized particles that are less likely to contribute effectively to SWCNT formation. In other words, W addition may improve the matching between the nanoparticle size distribution and the catalyst‐size window associated with SWCNT growth. Such an interpretation is consistent with recent FC‐CVD studies showing that CNT diameter is closely related to the catalyst particle size distribution and that the fraction of catalyst nanoparticles within the size range associated with CNT‐growing particles strongly affects the overall CNT production rate [87]. Consistent with this interpretation, the specific yield defined in Equation 2 in the Experimental Section increased by more than threefold upon W addition (W/Fe = 0.0: 1.4 ± 0.3 cm^2^ L^−1^; W/Fe = 0.2: 4.3 ± 0.4 cm^2^ L^−1^; W/Fe = 0.4: 5.1 ± 0.3 cm^2^ L^−1^), as shown in Figure S4.

The metallic fraction was 38.8% for the W‐free sample and approximately 33% for the W‐containing samples. Because these values are close to the random expectation of 1/3, we do not interpret this small change as evidence for intentional control of the metallic/semiconducting ratio. Instead, the metallic fraction is treated here as a descriptive parameter of the finite NBED‐analyzed population. The slight decrease in the relative metallic fraction may arise, at least in part, as a natural consequence of the W‐associated redistribution of chiralities toward the near‐armchair region. Although exact armchair (n,n) CNTs are metallic, ultra‐near‐armchair species such as (n,n−1) and (n,n−2) are semiconducting. Therefore, if W addition enriches near‐armchair species mainly through an increased population of such semiconducting near‐armchair CNTs, the apparent metallic fraction can decrease.

In addition to this distribution‐based effect, diameter‐ and metallicity‐dependent stability during growth may also contribute. In the present system, both methanol and citric acid contain oxygen, and their thermal decomposition in the reactor could generate oxygen‐containing species. Such oxidative species may preferentially etch metallic and/or smaller‐diameter CNTs, because metallic tubes have a lower ionization energy and are generally more reactive [88, 89], while smaller‐diameter CNTs possess larger curvature‐induced strain and lower chemical stability [90, 91]. Therefore, if W addition shifts the nucleated and grown CNT population toward smaller diameters, CNTs that combine both small diameter and metallic character may become particularly susceptible to etching by oxidative species derived from methanol and citric acid. This selective removal pathway could also contribute to a relative decrease in the metallic fraction in the final CNT ensemble [38, 92].

In principle, it cannot be entirely ruled out that W existed as independent nanoparticles, either as elemental W or as WC‐related species, rather than mixing with Fe, and that such W‐based particles directly catalyzed the growth of CNTs with structures distinct from those obtained from Fe‐based catalysts. To examine this possibility, we performed a control synthesis without ferrocene as the Fe source, using only AMT, citric acid, methanol, C_2_H_4_, H_2_, and N_2_. Under these conditions, however, we did not obtain a CNT network that could be dry‐transferred onto a substrate, which is the target product form in this study and requires a sufficient CNT number density and tube length to form a mechanically continuous film. This result implies that, under the present conditions, either CNT growth was negligible, the CNT number density was extremely low, or CNTs formed but remained too short to produce a percolated network. Although CNT growth from WC‐based catalysts has been reported in prior studies [35, 66], such syntheses have not yet demonstrated yields comparable to those obtained using Fe‐based catalysts, either in substrate‐based CVD or in FC‐CVD. In high‐yield CVD growth, transition metals with high carbon solubility and fast carbon diffusion, particularly Fe, Co, and Ni, are typically employed as the primary catalysts, whereas refractory metals such as W generally exhibit low carbon solubility and are therefore less likely to sustain comparable growth efficiencies without additional design considerations [93]. Taken together, the control experiment and the literature context suggest that the pronounced shifts in diameter, chiral‐angle distribution, and metallic fraction observed over the range W/Fe = 0–0.4 (Figure 3) are unlikely to be explained solely by a CNT subpopulation grown directly from W‐ or WC‐based particles as the dominant catalyst. We therefore interpret W primarily as an additive component that modifies the effective state and interfacial properties of the Fe‐based catalyst system, rather than as a dominant independent W‐ or WC‐based catalyst. In this interpretation, W influences the interfacial‐state parameter *γ*′ and thus *β*, and may also affect the active‐site‐related contribution reflected in *δ*, ultimately leading to the observed near‐armchair enrichment and diameter reduction.

### 2.2 Effect of Carbon Supply Rate on the CNT Chiral‐Angle Distribution in Mist FC‐CVD

In the previous section, we experimentally showed that introducing W‐containing species into an Fe‐based catalyst system in mist FC‐CVD shifted the CNT chiral‐angle distribution to a more sharply peaked profile concentrated in the near‐armchair region. We further showed that, based on the theoretical expression (Equation 1) and its parameters, this distribution change can be interpreted, at least in part, in terms of increased structural stability of the catalyst nanoparticles induced by W addition. An important next question, however, is whether similar changes in the chiral‐angle distribution can also be induced by process parameters that do not primarily act through thermal stabilization of the catalyst. Indeed, several previous studies [33, 39, 74, 94, 95] have reported near‐armchair‐enriched distributions even in Fe‐only catalyst systems without the addition of high‐melting‐point metals such as W. Because the chiral‐angle‐dependent abundance is theoretically expected to reflect not only interfacial thermodynamics but also growth kinetics that are sensitive to carbon availability [96, 97], we therefore focused on the carbon supply rate and carried out additional synthesis experiments using the C_2_H_4_ flow rate as an experimental control parameter.

**FIGURE 5:**
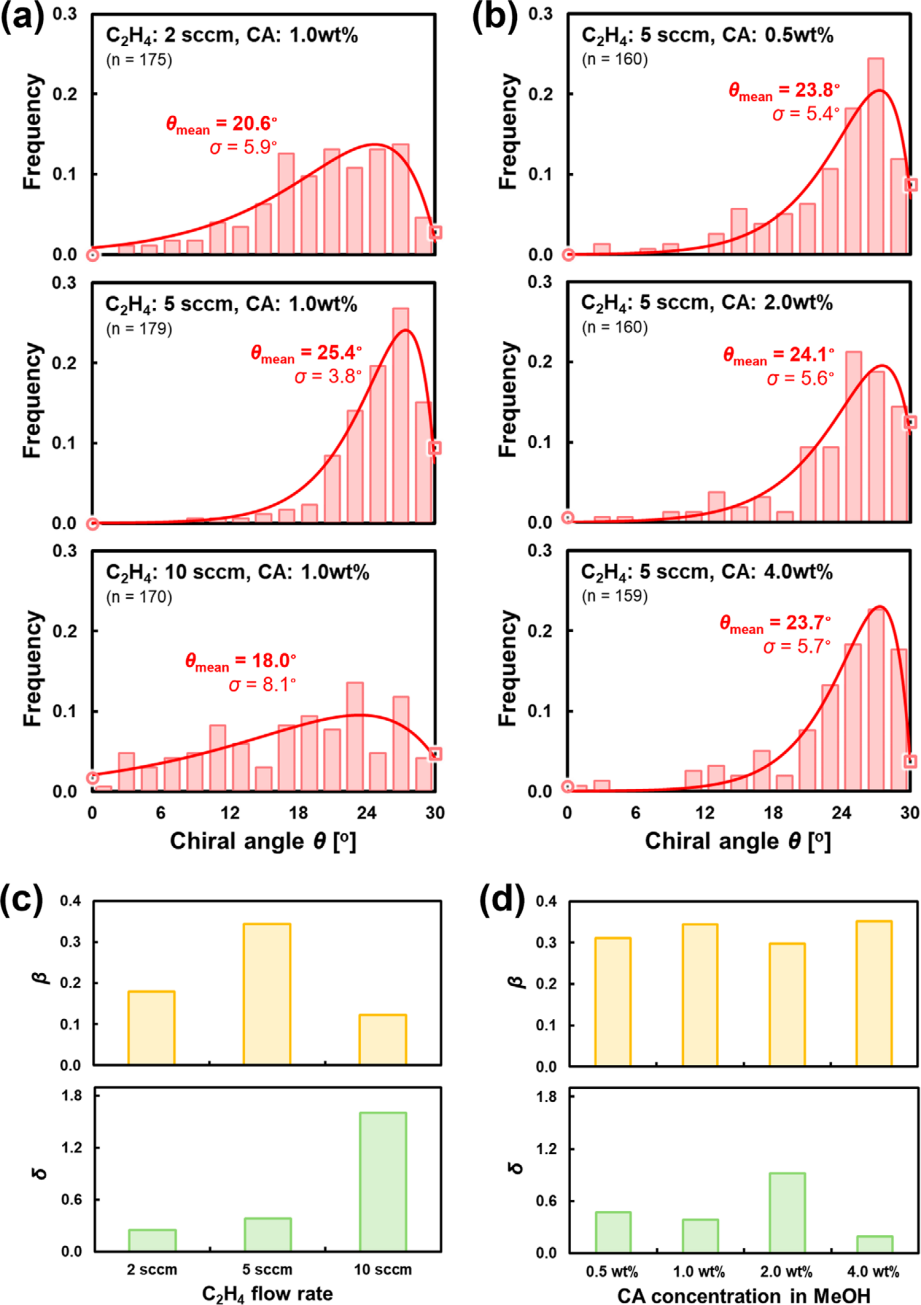
Chiral‐angle distributions (bin size: 2°) for CNTs synthesized at (a) different C_2_H_4_ flow rates and (b) different citric acid concentrations. Red solid lines show fits using Equation 1. Magenta circle and square markers at *θ* = 0° and 30° indicate the fractions of zigzag and armchair CNTs, respectively. Fitting parameters *β* and *δ* obtained from Equation 1 are summarized for (c) different C_2_H_4_ flow rates and (d) different citric acid concentrations.

Figure 5a summarizes the chiral‐angle distributions for different C_2_H_4_ flow rates, while Figure 5b shows the corresponding chiral‐angle distributions as a function of citric acid concentration. Representative TEM images of the CNT products synthesized under the C_2_H_4_‐series conditions are also provided in Figure S5 to show the overall morphology of the collected samples. The statistical parameters of the chiral‐angle distributions are listed in Tables S4 and S7. For the C_2_H_4_ series, a pronounced change in the chiral‐angle distribution was observed. At C_2_H_4_ = 2 sccm (*θ*_median_ = 21.2°, *θ*_mean_ = 20.6°, *σ* = 5.9°) and C_2_H_4_ = 10 sccm (*θ*_median_ = 19.1°, *θ*_mean_ = 18.0°, *σ* = 8.1°), the distributions were relatively broad and peaked toward lower chiral angles. In contrast, at C_2_H_4_ = 5 sccm, the distribution shifted toward higher angles close to the armchair limit and became notably sharper, with *θ*_median_ = 26.0°, *θ*_mean_ = 25.4°, and *σ* = 3.8°. This change in distribution shape is also reflected in the skewness and excess kurtosis values. The C_2_H_4_ = 5 sccm condition exhibited skewness = −1.49 and excess kurtosis = 3.35, indicating a sharply peaked distribution biased toward high chiral angles, whereas C_2_H_4_ = 2 sccm (skewness = −0.63, excess kurtosis = 0.00) and C_2_H_4_ = 10 sccm (skewness = −0.45, excess kurtosis = −0.79) showed broader, less sharply peaked distributions.

To verify that this trend was not an artifact of histogram binning, we performed pairwise nonparametric tests using the raw chiral‐angle values (Tables S5 and S6). Mann‐Whitney tests showed that the C_2_H_4_ = 5 sccm sample was significantly shifted toward higher chiral angles relative to both the 2 and 10 sccm samples. Two‐sample KS tests also confirmed that the overall empirical distribution at 5 sccm differed significantly from those at 2 and 10 sccm.

In contrast, varying the citric acid concentration produced no substantial change in the chiral‐angle distribution. Across the examined range, *θ*_median_ remained within 24.9–26.0°, *θ*_mean_ within 23.7–25.4°, and *σ* within 3.8–5.7°. Similarly, skewness (from −1.81 to −1.47) and excess kurtosis (from 2.48 to 3.79) varied only within a narrow window, and no systematic dependence on citric acid concentration was evident. Pairwise Mann‐Whitney and KS tests also detected no significant rank‐based shift or distributional difference after Bonferroni correction, supporting the absence of a systematic citric‐acid‐concentration dependence under the present conditions (Tables S8 and S9). These results indicate that the C_2_H_4_ flow rate can significantly alter the chiral‐angle distribution in mist FC‐CVD, whereas variation in citric acid concentration produced no measurable change under the present conditions.

We next discuss the potential mechanisms operating under each C_2_H_4_ condition. For C_2_H_4_ = 2 sccm, the chiral‐angle distribution remained relatively broad (*σ* = 5.9°, excess kurtosis = 0.00), and fitting with Equation 1 yielded *β* = 0.18 and *δ* = 0.25 (Table S4). The moderate *β* indicates that chiral‐angle selectivity was still present, but not strongly expressed under this condition. This behavior is consistent with a carbon‐supply‐limited regime, in which slow carbon adsorption and decomposition reduce growth‐rate contrasts among chiral angles, making it difficult for intrinsic chirality‐dependent kinetics to be amplified into a sharply peaked ensemble distribution [96]. Accordingly, compared with the optimal condition (C_2_H_4_ = 5 sccm), the 2 sccm condition resulted in a broader distribution and weaker near‐armchair enrichment.

For C_2_H_4_ = 5 sccm, the chiral‐angle distribution became markedly narrower, with *θ*_median_ = 26.0°, *θ*_mean_ = 25.4°, and *σ* = 3.8°, and the fitting yielded the largest *β* value of 0.34. The increased *β* indicates that the system moved beyond the carbon‐supply‐limited regime while avoiding the non‐ideal effects associated with overfeeding, allowing intrinsic chiral‐angle‐dependent kinetics to be expressed most effectively in the ensemble distribution. This interpretation is consistent with the general theoretical picture of CNT growth, in which nucleation thermodynamics and growth kinetics are predicted to jointly shape the abundance distribution, giving rise to a peak toward the near‐armchair region [47, 96].

For C_2_H_4_ = 10 sccm, the chiral‐angle distribution became broad again (*σ* = 8.1°, excess kurtosis = ‐0.79), but the fitted parameters were qualitatively different from those at 2 sccm. In this case, *β* decreased to 0.12, the smallest value among the tested conditions, while *δ* increased markedly to 1.60. These trends suggest that under the overfeeding conditions, the distribution is no longer governed solely by the idealized kink‐controlled chiral‐angle dependence, and that Equation 1 should therefore be interpreted as an effective description under this condition.

To further examine this interpretation, we measured the specific yield as a function of C_2_H_4_ flow rate (Figure S6). The yield increased up to approximately 7 sccm but decreased markedly at 10 sccm, even though the C_2_H_4_ feed rate was further increased. This non‐monotonic behavior suggests that productive CNT growth was suppressed under the 10 sccm condition, consistent with the onset of a non‐ideal carbon‐rich regime. Under such excessive‐carbon‐supply conditions, the catalyst surface can become carbon‐rich, and carbon incorporation may become less strictly constrained by the geometrically required kink density. In this regime, the growth edge may undergo dynamic reconstruction, and carbon atoms may be incorporated through multiple, less chirality‐sensitive pathways, including transient insertion events even when ideal kink sites are scarce. Such behavior would reduce the chiral‐angle contrast in *R*(*θ*) (lower effective *β*) while increasing the relative weight of angle‐insensitive baseline contributions captured by *δ* (higher *δ*). In addition, excess carbon can precipitate as graphitic or amorphous overcoats that progressively block active sites and eventually encapsulate the catalyst, leading to deactivation or premature termination [98]. These extrinsic processes introduce effects beyond those captured by a simple *A*(*θ*) ∝ *N*(*θ*)·*R*(*θ*) framework, which is consistent with the reduced *β*, enhanced *δ*, and the broadened distribution observed at 10 sccm.

Although both C_2_H_4_ = 2 and 10 sccm produced broad chiral‐angle distributions, the underlying mechanisms were different. At 2 sccm, the system was primarily carbon‐supply‐limited, so growth‐rate contrasts among chiral angles were not sufficiently expressed, resulting in a relatively broad distribution. In contrast, at 10 sccm, non‐ideal processes associated with overfeeding became prominent, leading to a deviation from the idealized kink‐controlled chiral‐angle dependence. By comparison, the intermediate condition of 5 sccm provided an optimal growth window in which supply‐limited growth was avoided while the effects of overfeeding were suppressed. As a result, the chiral‐angle dependence described by the theoretical abundance expression was manifested most strongly, yielding the narrowest distribution and the most pronounced bias toward the near‐armchair side. These results suggest that near‐armchair enrichment described by Equation 1 emerges clearly when kinetically appropriate growth conditions are satisfied. In addition, because no clear effect of citric acid concentration was observed within the examined range, citric acid is not considered to play a major role as an effective carbon source contributing to the kinetics that govern the chiral‐angle distribution in this mist FC‐CVD system.

From this perspective, the clear emergence of near‐armchair enrichment may require at least two conditions to be reasonably satisfied. First, the system should lie within an optimal growth window where productive CNT growth can proceed without being strongly limited by carbon starvation or overfeeding‐induced deactivation. This allows the chiral‐angle dependence described by *A* = *N* × *R* to emerge in the ensemble distribution. Second, once this prerequisite is met, a further increase in the effective thermal/structural stability of the catalyst system may shift the chiral‐angle distribution further toward the armchair side and sharpen it. In this sense, optimization of the carbon supply rate, as discussed in this section, can be regarded as a prerequisite for the emergence of chiral‐angle selectivity, whereas the stabilization‐based effect associated with W‐containing species, discussed in the previous section, can be understood as a factor that further sharpens the distribution once that prerequisite is met. It should also be noted that the establishment of this first condition may be influenced not only by carbon supply rate, but also by other process parameters, such as etching caused by water or oxygen contamination, etching under high H_2_ concentrations, deactivation caused by excessive sulfur supply, or insufficient decomposition of the carbon source.

## 3 Conclusions

In this study, near‐armchair‐enriched CNT growth was experimentally demonstrated in mist FC‐CVD, and comparison with a theoretical framework provided mechanistic insight into the factors governing the chiral‐angle distribution. By integrating ultrasonic aerosol‐mist generation, a non‐volatile W precursor (AMT) was co‐delivered with ferrocene into an atmospheric‐pressure FC‐CVD reactor, enabling the introduction of W‐containing species into an Fe‐based catalyst system in a continuous gas‐phase CNT synthesis process. Compared with W‐free growth, the introduction of W‐containing species was associated with a shift of the chiral‐angle distribution toward the near‐armchair region and a significant narrowing of the distribution.

Quantitative analysis based on a theoretical abundance expression for SWCNT growth on catalysts showed that W addition increased the angle‐selectivity parameter *β* while decreasing the additive term *δ*. These parameter changes are consistent with a stabilization‐based interpretation in which W‐containing species may increase the effective thermal/structural stability of the Fe‐based catalyst system and the catalyst–CNT interface, thereby strengthening the chiral‐angle dependence represented by *β* and suppressing baseline growth contributions near the achiral limits. Although the exact W‐containing active phase, chemical state, and alloying behavior remain unresolved, the observed trends are consistent with previous catalyst‐stabilization concepts involving refractory‐metal‐containing Fe‐ or Co‐based CNT‐growth catalysts.

In addition to catalyst design, the carbon supply rate was identified as a key process parameter governing the chiral‐angle distribution in mist FC‐CVD. Systematic variation of the C_2_H_4_ flow rate revealed an optimal growth window that maximized near‐armchair enrichment and minimized the distribution width, whereas both carbon‐supply‐limited and carbon‐rich conditions led to broader distributions, consistent with distinct kinetic regimes. These results indicate that near‐armchair enrichment in continuous FC‐CVD is favored by both an effective catalyst state suitable for selective growth and a carbon supply regime that allows the intrinsic chiral‐angle dependence of nucleation and growth to be expressed efficiently.

Overall, this work shows that mist FC‐CVD provides a practical route for introducing low‐volatility and non‐volatile metal precursors into continuous FC‐CVD growth, thereby expanding the accessible catalyst design space beyond conventional precursor limitations. The combination of W‐containing precursor design and carbon‐supply optimization provides practical design guidelines for chiral‐angle‐controlled CNT synthesis and offers a basis for further development of scalable structure‐controlled CNT production.

## 4 Experimental Section

### 4.1 CNT Synthesis in a Mist FC‐CVD Reactor

CNTs were synthesized using a vertical atmospheric‐pressure FC‐CVD reactor equipped with an ultrasonic nebulization unit (Figure 1a). The reactor consisted of an alumina tube (inner diameter 22 mm, outer diameter 28 mm) placed in a vertical tube furnace with a heating‐element length of 500 mm. Ferrocene (FeCp_2_, 99%, Alfa Aesar) was used as the Fe catalyst precursor, and thiophene (>99%, Sigma–Aldrich) was used as a growth promoter. Ferrocene and thiophene were first dissolved in methanol (MeOH, ≥99.9%, Sigma–Aldrich) at prescribed concentrations (ferrocene: 0.1 wt.% in methanol; thiophene: S/Fe = 1.0, defined as the molar ratio of sulfur atoms to iron atoms). For mist‐based delivery, the solvent must be readily nebulized, dissolve all precursors, and not strongly etch the as‐grown CNTs. Several candidate solvents, including acetone, toluene, and ethanol, were examined in preliminary experiments, and methanol was selected because it best satisfied these requirements. AMT ((NH_4_)_6_H_2_W_12_O_40_·*x*(H_2_O), 99.99%, Sigma–Aldrich) was used as the W precursor. Because AMT exhibits limited solubility in alcohol‐based solvents, citric acid (CA, ≥99.5%, Sigma–Aldrich) was dissolved in methanol (0.5–4 wt.%) and used as a chelating agent to stabilize W species. Unless otherwise stated, the citric acid concentration was fixed at 1 wt.%. Citric acid can coordinate with W(VI) to form tungsten citrate complexes, including reported mononuclear and dinuclear species, such as [W_2_O_5_(cit)_2_]^6−^ [99, 100, 101]. Such complexation suppresses aggregation and precipitation of W species, thereby improving their stability in solution. This strategy was partially inspired by the polymerized complex method [70, 102, 103], which employs chelating ligands such as citric acid to form homogeneous metal complexes prior to thermal conversion into oxide films or nanoparticles. In the conventional polymerized complex method, ethylene glycol is commonly used to promote esterification and polymerization prior to calcination. In the present study, however, ethylene glycol was intentionally omitted to avoid the possibility that ethylene‐glycol‐derived polymerized species would enhance the aggregation and coarsening of metal species after transport into the reactor and thermal decomposition, thereby favoring the formation of larger catalyst particles. Instead, the citric‐acid‐complexed precursor solution was directly delivered into the reactor as an ultrasonic mist to promote the formation of small catalyst particles suitable for SWCNT growth. To prepare the precursor solution, the ferrocene/thiophene/methanol solution and the AMT/citric acid/methanol solution were combined in a single vial and ultrasonicated for approximately 20 min (Figure 1b). The resulting precursor solution containing ferrocene, thiophene, AMT, citric acid, and methanol was sealed in a capped glass bottle equipped with a carrier‐gas inlet/outlet and placed in a water‐filled beaker serving as the ultrasonic propagation medium. The beaker was positioned above an ultrasonic generator operated at a fixed frequency of 1.7 MHz. The distance between the ultrasonic generator and the solution container was adjusted in advance to achieve stable mist generation, as confirmed visually. N_2_ (400 sccm, AGA, Finland), filtered through an oxygen/moisture purifier (OT3‐4, Agilent), was used as the carrier gas to transport the generated aerosol mist, and the solution supply rate was determined to be ∼160 µL min^−1^ based on preliminary calibration experiments. The aerosol‐mist‐containing N_2_ stream was merged with an additional N_2_/H_2_ stream (1000 sccm, 5% H_2_, Woikoski) and with C_2_H_4_ (99.995%, Linde Gas) as the carbon source. The combined gas mixture was then introduced into the reactor maintained at 1100°C. To suppress precursor re‐deposition and wall adsorption in the upstream transfer line, the stainless‐steel tubing (1/4‐inch outer diameter) connecting the gas‐delivery section to the reactor inlet was heated to 80°C using a heating tape. CNTs synthesized in the gas phase were collected at the reactor outlet using a nitrocellulose membrane filter (MF‐Millipore, pore size 0.45 µm) with an effective filtration diameter of 22 mm. The as‐grown CNT films on the filter were subsequently dry‐transferred onto glass slides for characterization following a previously reported method [13].

### 4.2 Transmission Electron Microscopy Analysis of CNTs and Catalyst Particles

HR‐TEM was employed to examine the structural features of CNTs, including bundle morphology and residual catalyst particles. CNTs were sampled directly from the FC‐CVD reactor onto a 200‐mesh lacey‐carbon‐coated copper grid placed on the membrane filter during collection. The prepared grids were then introduced into the high‐vacuum column of a double Cs‐corrected TEM (2200FS, JEOL, Japan). HR‐TEM imaging and EDS measurements were conducted at 200 kV. For EDS, spectra were acquired in TEM mode using a condensed electron beam positioned on selected regions of interest to identify catalyst‐derived elements.

NBED measurements were also performed on the same instrument and sample grids. To mitigate knock‐on damage to the CNT lattice, the accelerating voltage was reduced to 80 kV during diffraction acquisition. NBED patterns of individual SWCNTs were collected in nanobeam diffraction mode with an exposure time of approximately 10–20 s. The acquired diffraction patterns were analyzed using a calibration‐free TEM‐NBED chirality‐assignment protocol based on intrinsic layer‐line spacings, combining local candidate search with self‐consistency‐based cross‐check correction while explicitly accounting for manual measurement errors and CNT tilt [104, 105, 106]. The reliability of this procedure was previously evaluated using 2,394 simulated diffraction patterns [107], yielding an assignment accuracy of 97.7% [106].

Statistical analyses of the NBED‐derived chiral‐angle distributions were performed using the raw chiral‐angle values. Pairwise Mann‐Whitney tests were used to evaluate rank‐based shifts between synthesis conditions, and two‐sample KS tests were used to evaluate differences in the overall empirical distributions. For multiple pairwise comparisons within each series, Bonferroni correction was applied to the significance threshold. Bootstrap 95% confidence intervals for selected distribution parameters were calculated by resampling the raw NBED datasets with replacement using 10,000 resampling iterations.

### 4.3 Optical Characterization and Yield Evaluation of CNT Films

The optical properties of CNT films transferred onto glass substrates were characterized using a UV–Vis–NIR spectrometer (Cary 5000, Agilent Technologies, USA). Based on the optical transmittance at 550 nm (*T*) measured by UV–Vis–NIR spectroscopy, the specific CNT yield, defined as the transmittance‐derived equivalent CNT film area per unit carrier‐gas volume (cm^2^ L^−1^), was calculated according to Ref. [108] as follows:

(2) $$Specific\;yield=\frac{S_{f}\times \mathrm{lo}g_{0.9}\left(T\right)}{Q_{f}\times t}\;$$

where *S*_f_ is the effective filtration area (cm^2^), *Q*_f_ is the volumetric gas flow rate through the filter (L min^−1^), and *t* is the collection time (min).

In addition to this transmittance‐derived specific yield, a mass‐based production rate was evaluated for the W/Fe = 0.0 condition, which showed the lowest specific yield in the W/Fe series. CNT products were collected for an extended synthesis time under these conditions, and the mass of the collected CNTs was measured. The mass‐based production rate, calculated by dividing the collected CNT mass by the collection time, was determined to be 0.76 mg h^−1^.

To evaluate the structural characteristics of the CNTs, Raman spectroscopy was performed using a Raman spectrometer (Jobin–Yvon LabRAM HR800, Horiba, Japan) equipped with three excitation wavelengths: 488, 514, and 633 nm. All Raman spectra were normalized to the G band intensity.

## Conflicts of Interest

The authors declare no conflicts of interest.

## Supporting information




**Supporting File**: smll74316‐sup‐0001‐SuppMat.pdf.

## Data Availability

The data that support the findings of this study are available in the Supporting Information of this article.
