# Supplementary material for: Near‐Armchair‐Enriched Growth of Single‐Walled Carbon Nanotubes via W‐Containing Fe‐Based Catalyst Systems in Mist FC‐CVD
Source: Small. 2026 Jun 23;22(46):e74316. doi: 10.1002/smll.74316 (PMC13485023; doi:10.1002/smll.74316)
Supplement: Supplementary file 1 [file SMLL-22-e74316-s001.pdf]

Supporting Information

Near-Armchair-Enriched Growth of Single-Walled Carbon Nanotubes via W-Containing Fe-Based Catalyst Systems in Mist FC-CVD

Hirotaka Inoue[1,2*], Takamasa Onoki[2], Akira Takakura[2], Anastasios Karakassides[1], Hua Jiang[1], Toshihiko Fujimori[2,3], Esko I. Kauppinen[1*]

[1]Department of Applied Physics, School of Science, Aalto University, Puumiehenkuja 2, 02150 Espoo, Finland.

[2]Sumitomo Electric Industries, Ltd., 1-1-3 Shimaya, Konohana-ku, Osaka 554-0024, Japan.

[3]Institute of Applied Physics, University of Tsukuba, Tsukuba, 305-8573, Japan

[*]Corresponding author:

Hirotaka Inoue (inoue-hirotaka@sei.co.jp)

Esko I. Kauppinen (esko.kauppinen@aalto.fi)

Derivation of the Chiral-Angle Distribution Fitting Equation

To rationalize the observed changes in the chiral-angle distribution, we analyzed our data using the theoretical framework proposed by Artyukhov *et al.* [1] for SWCNT growth on solid catalysts. In this framework, the relative abundance of nanotubes with chiral angle θ and diameter d, $A(\theta,d)$, is expressed as the product of the thermodynamic nucleation probability $N(\theta,d)$ and the kinetic growth rate $R(\theta,d)$:

$$A(\theta, d) = N(\theta, d) \cdot R(\theta, d). \qquad \text{(Eq. S1)}$$

For nucleation, the free energy of the critical nucleus can be written as $G^* = G_{\text{cap}} + \pi d\gamma(\theta)$, where G_{cap} originates from the elastic energy of the cap and does not depend on θ, and $\gamma(\theta)$ is the effective edge energy associated with maintaining tight contact between the nanotube rim and the catalyst terrace. Consequently, the angular dependence of the nucleation term can be written as

$$N(\theta, d) \propto e^{-\frac{\pi d\gamma(\theta)}{k_{\text{B}}T}}, \qquad \text{(Eq. S2)}$$

where k_{B} is the Boltzmann constant and T is the temperature. Near an achiral direction, the deviation x from that achiral limit is introduced as $x = \theta$ (near zigzag) or $x = 30°-\theta$ (near armchair). In this limit, $\gamma(\theta)$ can be linearized as $\gamma(x) \approx \gamma_0 + \gamma'\cdot x$, so that constant factors may be absorbed into a prefactor and the nucleation probability reduces to an exponential decay with the angular deviation:

$$N(\theta) \propto e^{-\beta\cdot x}, \qquad \beta = \frac{\pi d\gamma'}{k_{\text{B}}T}. \qquad \text{(Eq. S3)}$$

The growth-rate term $R(\theta,d)$ is governed by the density of active incorporation sites (kinks) at the nanotube rim. In the solid-catalyst regime, the geometric kink density increases approximately

linearly with the angular deviation x, while additional "fluctuational kinks" can be created at achiral rims via thermal fluctuations. Incorporating the curvature penalty for small diameters, the growth rate can be expressed as

$$R(\theta, d) \propto \pi d \cdot e^{-\frac{2C}{d^2 k_B T}} \cdot \left(x + e^{-\frac{E}{k_B T}}\right), \qquad \text{(Eq. S4)}$$

where C is the bending rigidity of graphene and E is the free-energy barrier associated with initiating a new atomic row on an achiral rim. Defining $\delta = \exp(-E/k_B T)$ and assuming that d varies only within a sufficiently narrow range, the leading θ-dependence of the growth term is simplified to

$$R(\theta) \propto x + \delta. \qquad \text{(Eq. S5)}$$

Combining the simplified angular dependences of the nucleation term (Eq. S3) and the growth term (Eq. S5) yields the chiral-angle dependence of the abundance:

$$A(\theta) \propto (x + \delta) \cdot e^{-\beta \cdot x}. \qquad \text{(Eq. S6)}$$

Because our samples exhibit an armchair-side preference, we adopt $x = 30° - \theta$ and introduce a proportionality constant A_0, giving the final fitting form:

$$A(\theta) = A_0[(30° - \theta) + \delta] \cdot e^{-\beta(30° - \theta)}. \qquad \text{(Eq. S7)}$$

This expression is identical to Eq. 1 in the main text.

Table S1. Statistical parameters of chiral-angle distributions and fitting parameters obtained using Eq. 1 for CNTs synthesized with different W/Fe ratios.

W/Fe	Analyzed results from raw data					Fitting parameters		
	**θ_{median}** [°]	**θ_{mean}** [°]	**σ** [°]	**Skew ness** (norm. dist.: 0)	**Excess kurtosis** (norm. dist.: 0)	**A_0**	**β**	**δ**
0.0 (n=170)	19.1 *16.1 – 19.8	18.0 *16.8 – 19.8	8.1 *7.4 – 8.8	-0.45 *-0.68 – -0.23	-0.79 *-1.09 – -0.37	0.03	0.12	1.60
0.2 (n=160)	25.7 *25.0 – 26.3	24.4 *23.6 – 25.1	5.1 *4.3 – 6.0	-1.59 *-1.97 – -1.31	2.57 *1.19 – 4.54	0.13	0.30	0.76
0.4 (n=160)	25.1 *24.2 – 26.0	24.4 *23.6 – 25.1	4.6 *4.0 – 5.5	-1.40 *-1.92 – -1.01	2.59 *1.12 – 5.37	0.13	0.29	0.60

*Bootstrap 95% confidence intervals (Number of bootstrap samples: 10000, Method: BCa)

Table S2. Pairwise Mann-Whitney test results for the NBED-derived chiral-angle distributions in the W/Fe series.

Comparison **W/Fe**		Mann-Whitney Test					
		**Mean rank**					
group 1	group 2	group 1	group 2	***U***	***Z***	***p*-value**	***Interpretation**
0.2 (n=160)	0.0 (n=170)	**207.3**	**126.2**	20288	7.72	**< 0.0001**	W/Fe = 0.2 shifted toward higher θ
0.4 (n=160)	0.0 (n=170)	**206.6**	**126.9**	20168	7.59	**< 0.0001**	W/Fe = 0.4 shifted toward higher θ
0.4 (n=160)	0.2 (n=160)	157.7	163.3	12357	-0.59	0.5924	No significant difference was detected

*Interpretations are based on the Bonferroni-corrected significance threshold, α/m = 0.05/3 = 0.0167

Table S3. Pairwise Kolmogorov-Smirnov test results for the NBED-derived chiral-angle distributions in the W/Fe series.

Comparison **W/Fe**		Kolmogorov-Smirnov (KS) Test			
group 1	group 2	***D***	***Z***	***p*-value**	***Interpretation**
0.2 (n=160)	0.0 (n=170)	0.42	3.84	**< 0.0001**	Distributions differ
0.4 (n=160)	0.0 (n=170)	0.41	3.72	**< 0.0001**	Distributions differ
0.4 (n=160)	0.2 (n=160)	0.08	0.73	0.5999	No significant distributional difference was detected

*Interpretations are based on the Bonferroni-corrected significance threshold, α/m = 0.05/3 = 0.0167

Table S4. Statistical parameters of chiral-angle distributions and fitting parameters obtained using Eq. 1 for CNTs synthesized with an Fe monometallic catalyst at different C_2H_4 flow rates.

	Analyzed results from raw data					Fitting parameters		
**C_2H_4** [sccm]	**θ_{median}** [°]	**θ_{mean}** [°]	**σ** [°]	**Skew ness** (norm. dist.: 0)	**Excess kurtosis** (norm. dist.: 0)	**A_0**	**β**	**δ**
2 (n=175)	21.2 *20.2 – 22.2	20.6 *19.7 – 21.5	5.9 *5.4 – 6.6	-0.63 *-0.92 – -0.39	0.00 *-0.51 – 0.77	0.06	0.18	0.25
5 (n=179)	26.0 *25.3 – 26.6	25.4 *24.8 – 25.9	3.8 *3.3 – 4.6	-1.49 *-2.08 – -1.00	3.35 *1.30 – 6.70	0.20	0.34	0.38
10 (n=170)	19.1 *16.2 – 19.8	18.0 *16.8 – 19.2	8.1 *7.4 – 8.8	-0.45 *-0.68 – -0.22	-0.79 *-1.10 – -0.38	0.03	0.12	1.60

*Bootstrap 95% confidence intervals (Number of bootstrap samples: 10000, Method: BCa)

Table S5. Pairwise Mann-Whitney test results for the NBED-derived chiral-angle distributions in the C_2H_4 series.

Comparison		Mann-Whitney Test					
**C_2H_4 [sccm]**		**Mean rank**					
group 1	group 2	group 1	group 2	***U***	***Z***	***p*-value**	***Interpretation**
5 (n=179)	2 (n=175)	**221.2**	**132.8**	23485	8.13	**< 0.0001**	C_2H_4 = 5 sccm shifted toward higher θ
5 (n=179)	10 (n=170)	**223.4**	**124.1**	23877	9.20	**< 0.0001**	C_2H_4 = 5 sccm shifted toward higher θ
2 (n=175)	10 (n=170)	**187.2**	**158.4**	17357	2.68	**0.0074**	C_2H_4 = 2 sccm shifted toward higher θ

*Interpretations are based on the Bonferroni-corrected significance threshold, α/m = 0.05/3 = 0.0167

Table S6. Pairwise Kolmogorov-Smirnov test results for the NBED-derived chiral-angle distributions in the C_2H_4 series.

Comparison		Kolmogorov-Smirnov (KS) Test			
**C_2H_4 [sccm]**					
group 1	group 2	***D***	***Z***	***p*-value**	***Interpretation**
5 (n=179)	2 (n=175)	0.41	3.81	**< 0.0001**	Distributions differ
5 (n=179)	10 (n=170)	0.48	4.52	**< 0.0001**	Distributions differ
2 (n=175)	10 (n=170)	0.20	1.84	**0.0018**	Distributions differ

*Interpretations are based on the Bonferroni-corrected significance threshold, α/m = 0.05/3 = 0.0167

Table S7. Statistical parameters of chiral-angle distributions and fitting parameters obtained using Eq. 1 for CNTs synthesized with an Fe monometallic catalyst at different citric acid concentrations.

CA [wt%]	Analyzed results from raw data					Fitting parameters		
	θ_{median} [°]	θ_{mean} [°]	σ [°]	Skewness (norm. dist.: 0)	Excess kurtosis (norm. dist.: 0)	A_0	β	δ
0.5 (n=160)	24.9 *24.0 – 25.9	23.8 *22.9 – 24.5	5.4 *4.7 – 6.5	-1.47 *-1.98 – -1.06	2.48 *0.81 – 5.15	0.15	0.31	0.47
1.0 (n=179)	26.0 *25.3 – 26.6	25.4 *24.8 – 25.9	3.8 *3.3 – 4.6	-1.49 *-2.08 – -1.00	3.35 *1.30 – 6.70	0.20	0.34	0.38
2.0 (n=160)	25.3 *24.2 – 25.5	24.1 *23.2 – 24.9	5.6 *4.8 – 6.9	-1.72 *-2.30 – -1.29	3.48 *1.48 – 7.05	0.12	0.30	0.92
4.0 (n=159)	24.9 *24.1 – 25.7	23.7 *22.7 – 24.5	5.7 *4.8 – 7.0	-1.81 *-2.33 – -1.37	3.79 *1.84 – 7.09	0.21	0.35	1.19

*Bootstrap 95% confidence intervals (Number of bootstrap samples: 10000, Method: BCa)

Table S8. Pairwise Mann-Whitney test results for the NBED-derived chiral-angle distributions in the citric acid series.

Comparison CA [wt%]		Mann-Whitney Test					
group 1	group 2	Mean rank group 1	Mean rank group 2	U	Z	p-value	*Interpretation
0.5 (n=160)	1.0 (n=179)	156.5	182.1	12156	-2.40	0.0162	No significant difference was detected
0.5 (n=160)	2.0 (n=160)	156.1	164.9	12090	-0.86	0.3905	No significant difference was detected
0.5 (n=160)	4.0 (n=159)	160.2	159.8	12744	0.03	0.9772	No significant difference was detected
1.0 (n=179)	2.0 (n=160)	176.9	162.3	15553	1.37	0.1710	No significant difference was detected
1.0 (n=179)	4.0 (n=159)	181.7	155.8	16416	2.44	0.0148	No significant difference was detected
2.0 (n=160)	4.0 (n=159)	164.6	155.3	13462	0.90	0.3681	No significant difference was detected

*Interpretations are based on the Bonferroni-corrected significance threshold, α/m = 0.05/6 = 0.0083

Table S9. Pairwise Kolmogorov-Smirnov test results for the NBED-derived chiral-angle distributions in the citric acid series.

Comparison CA [wt%] group 1	Comparison CA [wt%] group 2	Kolmogorov-Smirnov (KS) Test *D*	*Z*	*p*-value	*Interpretation
0.5 (n=160)	1.0 (n=179)	0.14	1.30	0.0566	No significant distributional difference was detected
0.5 (n=160)	2.0 (n=160)	0.07	0.61	0.7851	No significant distributional difference was detected
0.5 (n=160)	4.0 (n=159)	0.06	0.50	0.9340	No significant distributional difference was detected
1.0 (n=179)	2.0 (n=160)	0.11	0.97	0.2607	No significant distributional difference was detected
1.0 (n=179)	4.0 (n=159)	0.13	1.20	0.0958	No significant distributional difference was detected
2.0 (n=160)	4.0 (n=159)	0.10	0.89	0.3521	No significant distributional difference was detected

*Interpretations are based on the Bonferroni-corrected significance threshold, α/m = 0.05/6 = 0.0083

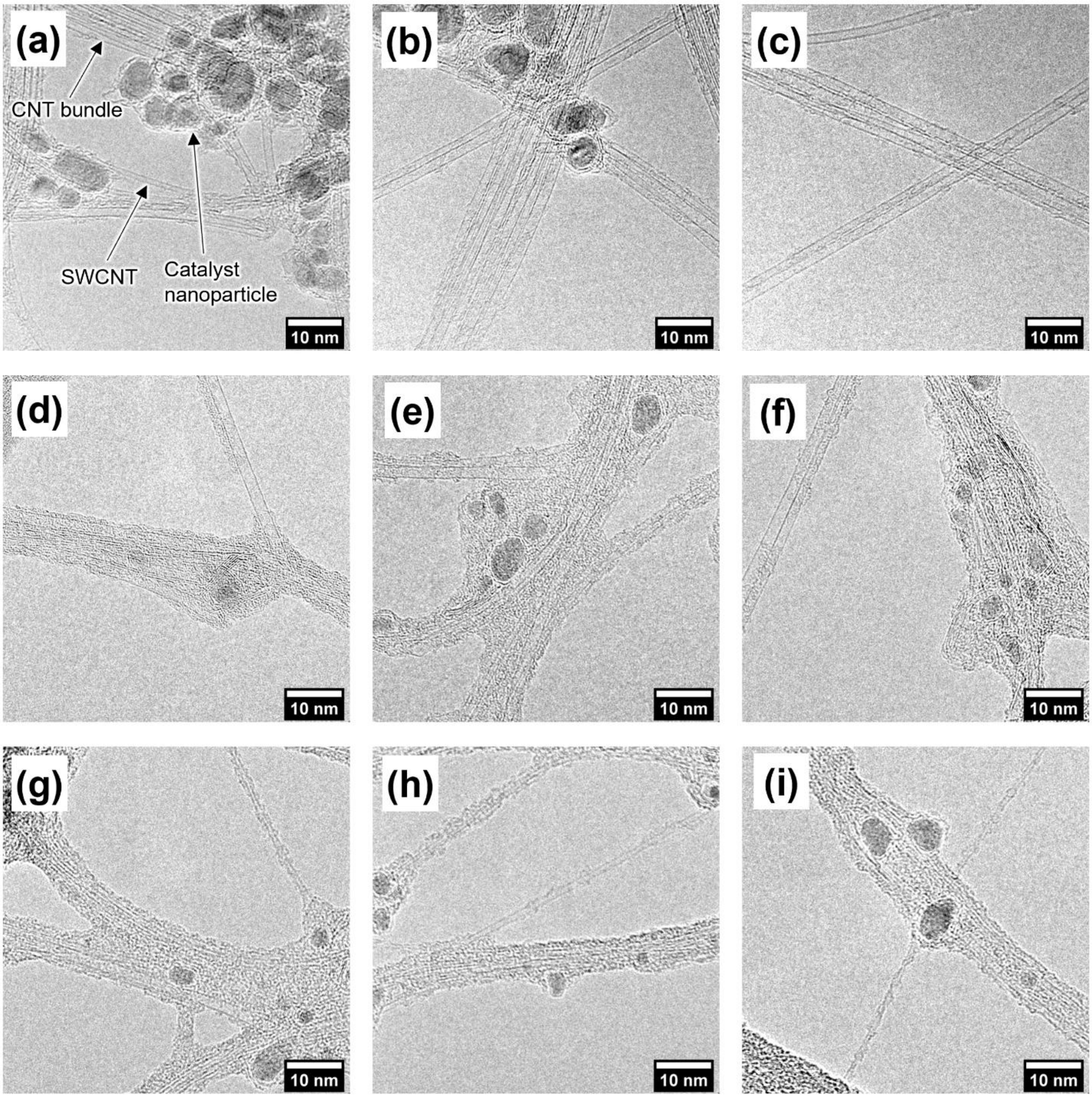

Figure S1. Representative post-growth TEM images of CNT products synthesized at different W/Fe ratios. (a–c) W/Fe = 0.0, (d–f) W/Fe = 0.2, and (g–i) W/Fe = 0.4.

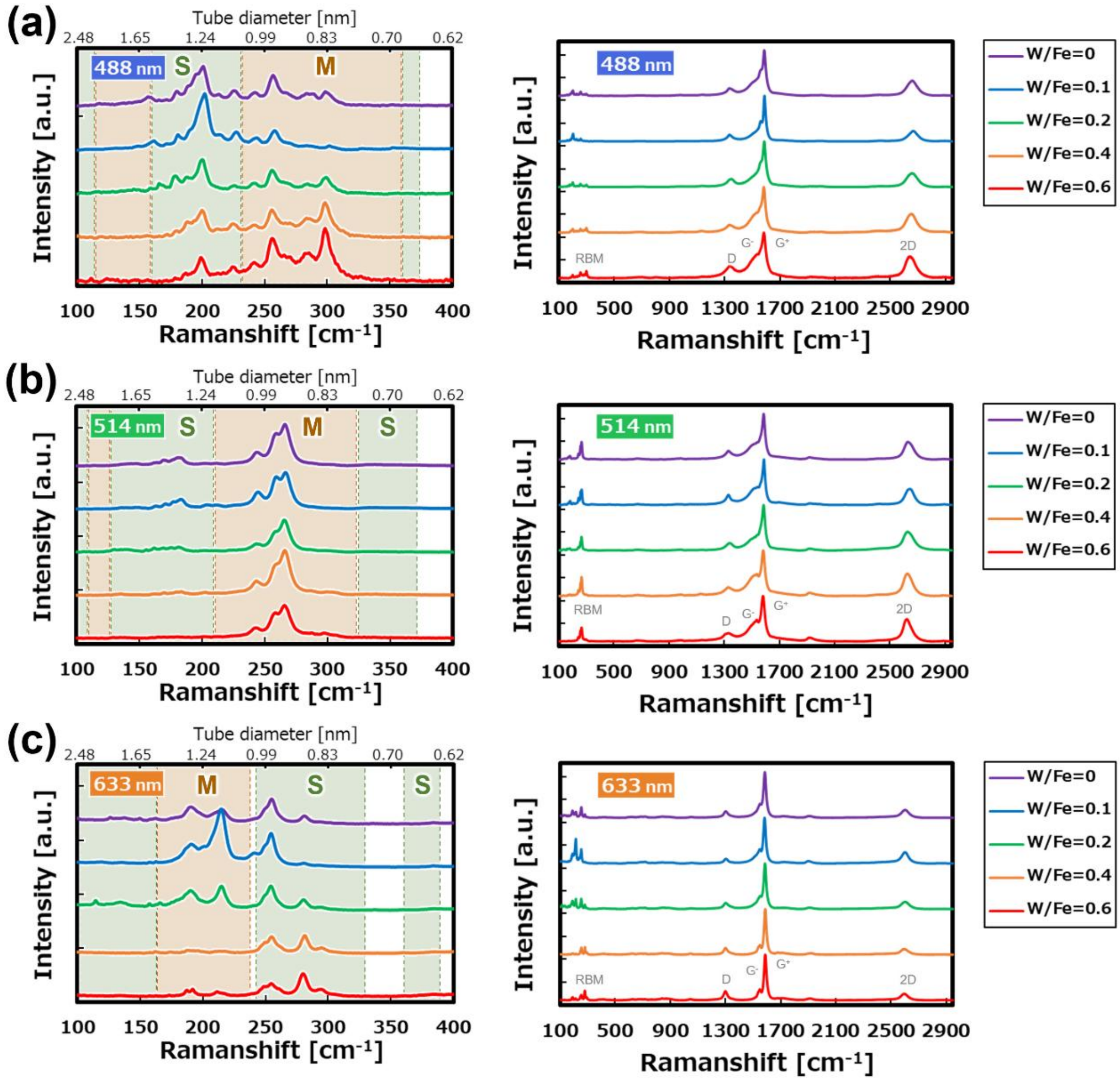

Figure S2. Raman spectra of CNTs synthesized at different W/Fe ratios, measured using excitation wavelengths of (a) 488, (b) 514, and (c) 633 nm. The right panels show the wide-range spectra, and the left panels show expanded views of the low-frequency region highlighting the RBM peaks. The brown-shaded region corresponds to metallic CNTs (M), whereas the green-shaded region corresponds to semiconducting CNTs (S).

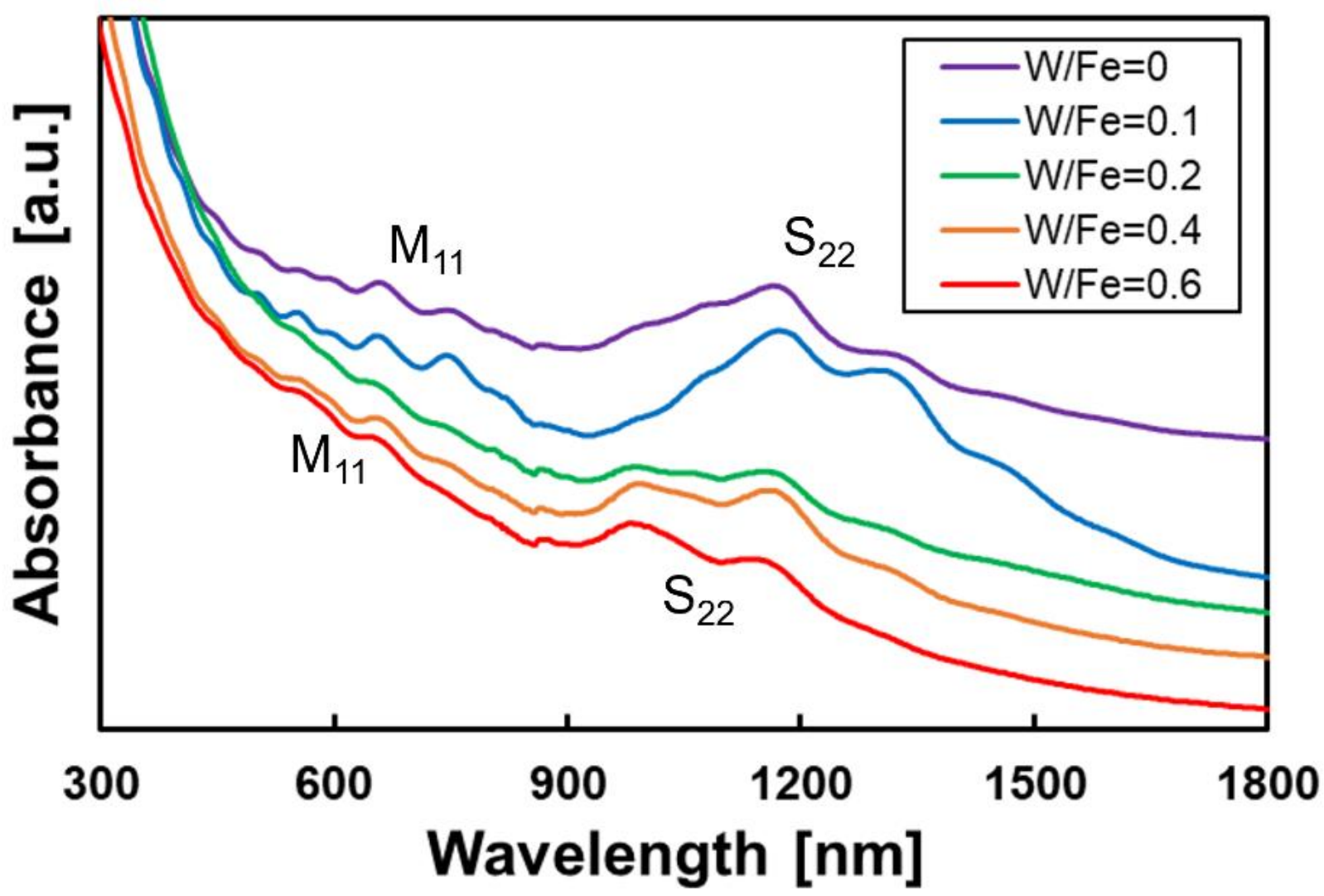

Figure S3. UV-Vis-NIR absorption spectra of CNT films synthesized under different W/Fe ratios.

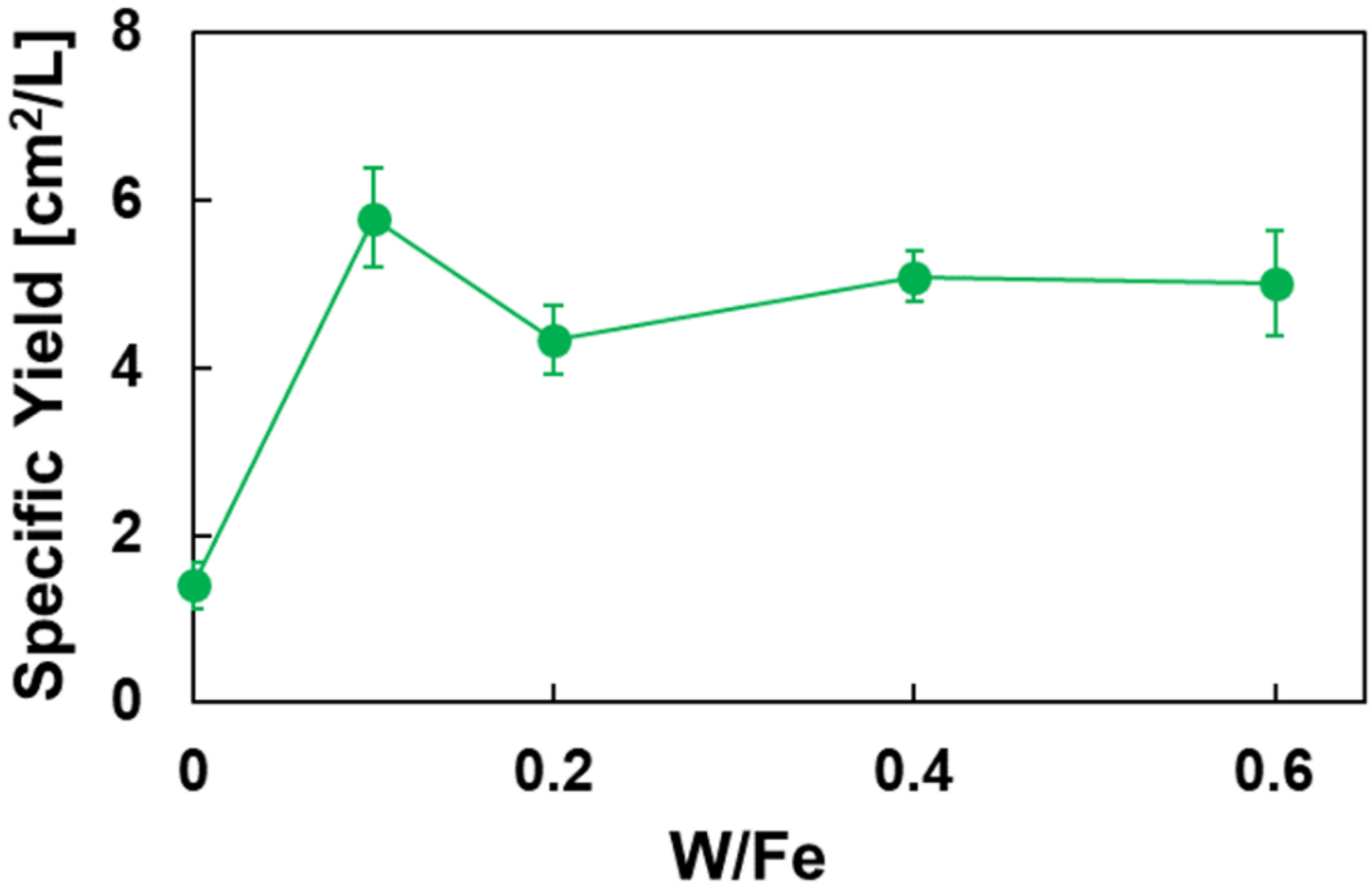

Figure S4. Specific yield of CNT films as a function of the W/Fe ratio.

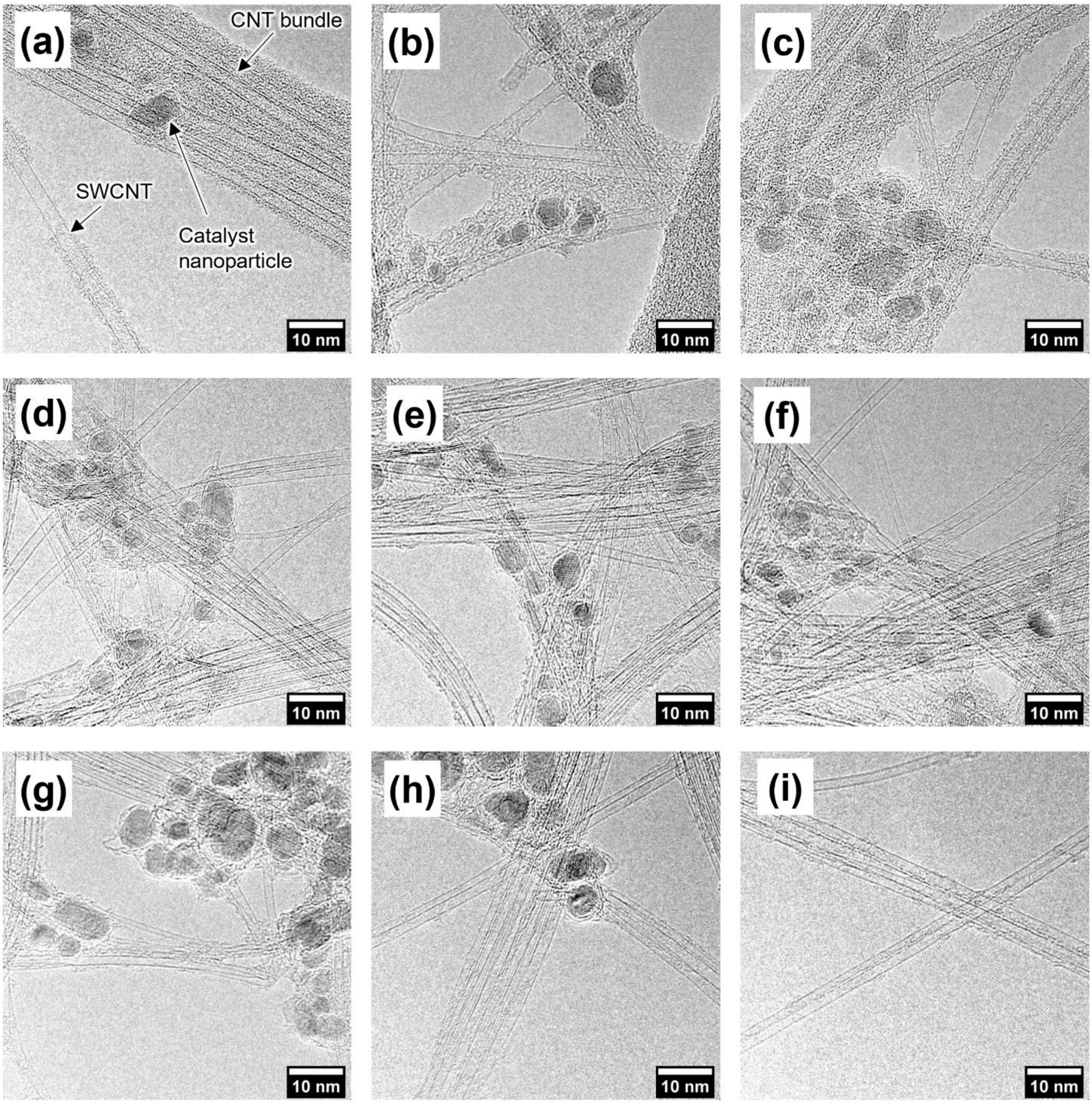

Figure S5. Representative post-growth TEM images of CNT products synthesized at different C_2H_4 flow rates. (a–c) C_2H_4 = 2 sccm, (d–f) C_2H_4 = 5 sccm, and (g–i) C_2H_4 = 10 sccm.

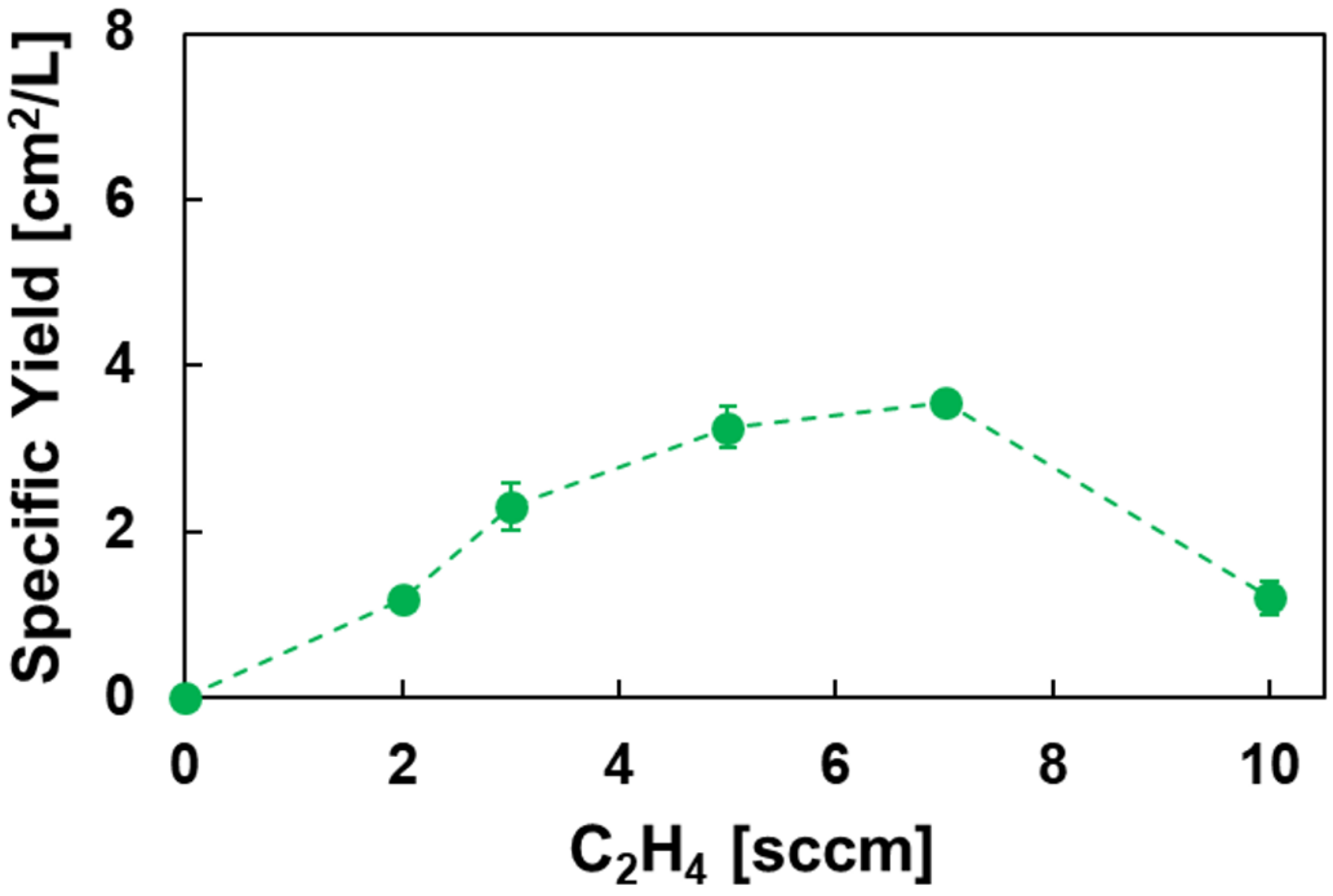

Figure S6. Specific yield of CNT films as a function of the C_2H_4 flow rate.

Supplementary References

[1] V. I. Artyukhov, E. S. Penev, B. I. Yakobson, "Why nanotubes grow chiral." *Nat. Commun.* **5** (1), 4892 (2014). https://doi.org/10.1038/ncomms5892.
